# Relative-Entropy Variational Principle for Semiclassical Gravity with Finite-Resolution Boundaries

**DOI:** 10.3390/e28060606

**Published:** 2026-05-28

**Authors:** Olivier Nusbaumer

**Affiliations:** Independent Researcher, 5330 Bad Zurzach, Switzerland; olivier.nusbaumer@alumni.ethz.ch

**Keywords:** quantum gravity, semiclassical gravity, entropic gravity, relative entropy, causal diamonds, finite-resolution boundaries, boundary-completed algebras, Wheeler–DeWitt equation, modular flow, Kubo–Mori metric

## Abstract

This work formulates semiclassical gravity within a causal-diamond framework where a finite-resolution boundary provides the edge structure for a local Wheeler–DeWitt description. Because the diffeomorphism-invariant Hilbert space does not factorize, each diamond is equipped with a boundary-completed algebra $A_{O}$, ensuring the operational state $\rho_{O}$ and the semiclassical reference family $\sigma_{O}\left[\Lambda \right]$ share identical operator content. Dynamics are posed as local statistical inference: the relative-entropy functional $S_{\mathrm{rel}}\left(\rho_{O}∥\sigma_{O}\left[\Lambda \right]\right)$ quantifies the mismatch between data and reference. This yields the minimal operational axioms defining subsystems, intrinsic clocks, and regulated observables in a finite-resolution, background-independent setting. The topology-locked boundary capacity budget fixes an effective channel multiplicity $N\approx 1.23\times 10^{11}$. Calibrating its coherent fraction to Newton’s constant determines a matching scale $M_{s}\approx 3.02\times 10^{13}\;\mathrm{GeV}$. In the modular/KMS regime, the relative-entropy Hessian (Kubo–Mori metric) block-diagonalizes into orthogonal tensor, vector, and scalar response sectors. A heat-kernel expansion on the fixed $S^{3}\times S^{1}$ history manifold maps this near-equilibrium response to a matching-scale effective field theory, yielding the Einstein–Hilbert tensor structure, Yang–Mills susceptibilities, and leading mass deformations. Vector and scalar responses remain intensive, while the tensor response scales extensively with coherent channel multiplicity. The fixed modular protocol and quantized boundary currents imply $\alpha^{-1}\left(M_{s}\right)=4\pi k$ at integer levels *k*, while the reduced $R^{2}$ plateau sector yields linked cosmological targets: $n_{s}≃0.965$, r≃0.0038, and $A_{s}≃2.1\times 10^{-9}$. Translations between causal diamonds act as completely positive trace-preserving (CPTP) updates. The resulting open-modular Walsh filtration selects the three-dimensional degree-one sector as the algebraic basis for family structure. Treating continuum fields as the structured response of a finite boundary, the framework yields correlated, falsifiable relations for gravitational stiffness, gauge response, plateau cosmology, and threefold matter-sector organization from one minimal operational architecture.

## 1. Conceptual Foundations

We propose a finite-resolution framework for defining local quantum subsystems in semiclassical gravity, organized around the Wheeler–DeWitt (WDW) constraint [1]:

$$\hat{H}\Psi =0$$

where the global state Ψ is constrained rather than time-evolved, and the operator $\hat{H}$ encodes gravitational constraints and diffeomorphism invariance.

To ensure background independence, we treat dynamics as local statistical inference on causal diamonds O(p,q) [2], the fundamental units of accessible correlations [3]. In the small-diamond regime, modular flow supplies the intrinsic local clock [4,5]. Geometry is inferred from an entropic mismatch functional between the actual boundary-completed state $\rho_{O}$ and a semiclassical reference family $\sigma_{O}\left[\Lambda \right]$ defined by the background fields Λ [6,7,8,9,10].

The minimal architecture consists of a causal diamond equipped with boundary completion that stores the edge data required for a subregion algebra [2,11]. The resulting topological capacity functional defines an effective internal degeneracy *N*, and calibration to Newton’s constant *G* fixes the matching-scale resolution $M_{s}$.

### 1.1. Axiomatic Basis: Minimal Architecture

In a background-independent theory, there is no external clock and no pre-existing global stage on which local observables are simply placed. Locality must therefore be defined operationally from the finite record accessible to an observer. The causal diamond provides the minimal covariant laboratory for this local inference [2], defined by two timelike-separated events and independent of global backgrounds. Within this region, diffeomorphism and Gauss-law constraints obstruct naive bulk factorization, so subregion data must be tied to a closed boundary interface, the waist $S^{2}$. Holographic bounds require finite information capacity. Modular flow then supplies the intrinsic clock, boundary completion defines the measurable algebra, and finite resolution fixes the common operator content used to compare $\rho_{O}$ with $\sigma_{O}\left[\Lambda \right]$.

Consequently, we formulate quantum gravity as local statistical inference in a background-independent, finite-resolution setting, bypassing the need for a preferred lattice or pre-defined field content.

The axioms P1–P7 define the operational domain for subsystems, clocks and regulated observables. Topology, transport and spin structure then specify the minimal boundary sector, while constitutive relations calibrate its microscopic capacity to macroscopic gravity. Phenomenological results follow as outputs of this combined architecture. The axioms are organized under three architectural principles:

Principle A: Operational Locality and Boundary Completion

Locality is defined operationally by the minimal covariant unit: a finite causal diamond [2]. This geometry fixes the boundary interface where subsystem-defining information must reside [11].

**P1 (Non-factorization)**. The diffeomorphism-invariant physical Hilbert space does not factorize across spatial subregions ($H_{\mathrm{phys}}\neq H_{A}⊗H_{B}$). Constraint relations tie interior data to boundary charges and fluxes, requiring explicit boundary data (edge modes) to define a well-specified subregion algebra [11,12,13,14].

**P2 (Causal Diamonds)**. A finite experiment is a closed query–response loop between two timelike-separated events *p* and *q*. A causal diamond $O\left(p,q\right)\equiv J^{+}\left(p\right)\cap J^{-}\left(q\right)$ [2] covariantly defines an observer’s operational workspace. We restrict to the minimal simply connected sector: a maximal spatial slice of topology $B^{3}$ with a single closed waist $S^{2}$. This is the minimal local topology supporting a closed boundary completion (P3); nontrivial topologies require extra gluing data and lie outside the present analysis.

**P3 (Boundary Completion)**. Since the physical Hilbert space does not factorize (P1), a well-defined subregion algebra $A_{O}$ requires boundary completion. States are positive, normalized functionals on $A_{O}$. This interface acts as a coherency screen at the diamond waist $S^{2}$, reconciling overlapping past and future boundary data into a unified record. We adopt a boundary-completed algebra whose finite-resolution center $Z(A_{O})$ carries the gluing and charge labels needed to encode Gauss-law constraints. This ensures that $\rho_{O}$ and $\sigma_{O}\left[\Lambda \right]$ live on identical operator content and define a common variational domain [11,13,15].

Principle B: Finite Modular Resolution

Finite capacity prevents unbounded boundary information density [16,17]. In the small-diamond KMS regime, modular flow supplies the intrinsic local clock [4,5,18].

**P4 (Modular Locality)**. In the local Rindler regime, the vacuum restricted to $A_{O}$ is approximately KMS with respect to a geometric modular flow. This applies in the small-diamond regime, where the diamond size is intermediate between the resolution limit and the local curvature radius. The modular Hamiltonian is well-approximated by the local boost generator, fixing KMS periodicity 2π [4,5].

**Corollary (Canonical History Manifold)**. In the minimal simply connected sector, the two spatial $B^{3}$ domains associated with the causal-diamond halves glue across the waist as $B^{3}\cup_{S^{2}}B^{3}≃S^{3}$. The KMS condition evaluates local response on a compact Euclidean modular cycle $S^{1}$. The spectral trace is therefore represented on $S^{3}\times S^{1}$, the minimal compact history manifold for this sector.

**P5 (Finite Resolution)**. Defining a local subsystem requires finite resolution to ensure a stable restriction to $A_{O}$ and a bounded mode density at the interface. Since an algebraic restriction alone cannot fix the physical bandwidth, a proper-distance cutoff $L_{s}\equiv M_{s}^{-1}=\delta$ is introduced via a stretched screen in the local Rindler region. This finite-capacity regulator guarantees that $\rho_{O}$ and $\sigma_{O}\left[\Lambda \right]$ admit a well-defined relative-entropy comparison, consistent with holographic bounds [16,19,20].

**Corollary (Monotone Spectral Suppression)**. Relative entropy is monotone under the restriction of the accessible algebra (P3), while finite resolution imposes a strict limit on the information capacity per pixel (P5). In the resolved quasi-local regime, the infrared expansion is therefore organized in terms of intensive response densities and irrelevant corrections suppressed by powers of $E/M_{s}$. Extensive macroscopic quantities may grow with the total number of pixels, but the distinguishability available to each pixel remains bounded by the finite-capacity algebra.

Principle C: Quantized Boundary Topology and Minimal Transport

Non-factorization (P1) and boundary completion (P3) place charge labels on the boundary, represented here as discrete topological sectors [21]. Finite resolution supplies an isotropic transport layer for local excitations [22]. Near the sub-resolution tips, smooth modular flow must be routed through discrete boundary updates; the resulting digitization cost is the bottleneck that limits phase-coherent capacity.

**P6 (Gauge Topology)**. Boundary charge sectors are encoded by topological current data compatible with non-factorization (P1) and closed algebraic gluing (P3). During one modular cycle, the causal-diamond waist sweeps out the closed boundary history $S^{2}\times S^{1}$, whose current response is represented by a Chern–Simons functional [21]. With the trace normalization fixed, large gauge transformations of compact simple groups shift this functional by integer multiples of 2πk; path-integral phase invariance, therefore, requires k∈Z [23]. Gauss law makes interior flux end on the spatial $S^{2}$ interface as representation-carrying punctures. These punctures generate surface states described by Wess–Zumino–Witten conformal blocks, equivalently chiral data organized by the affine Kac–Moody algebra at level *k* [24]. Retaining sectors compatible with KMS periodicity, large-gauge invariance, and anomaly inflow, this integer level fixes the boundary-current normalization entering the matching-scale inverse gauge coupling.

**P7 (Discrete Isotropic Transport)**. A finite-resolution boundary architecture requires a local transport layer that is isotropic after modular averaging, inversion-symmetric, and closed under frame transport. Modular closure requires that after a 2π period, local excitations return to their initial states up to a minimal grading, lacking spurious phases or directional bias. Inversion symmetry pairs every transport direction with its opposite. The absence of directional bias requires the modularly averaged transport to be isotropic, so the second moment of the local step distribution is proportional to the identity. For three equal-length antipodal pairs with equal weights, this condition forces orthonormality, fixing the minimal transport layer to the signed basis {±x,±y,±z}, octahedral coordination z=6, and the $L_{1}$ step rule. This closure requires lifting SO(3) to its double cover SU(2), introducing a minimal $Z_{2}$ grading, and thereby supporting local spinors. Since finite resolution (P5) sets the only intrinsic scale, we select a massless continuum transport operator that recovers local conformal covariance, removing curvature-coupling ambiguities.

**Corollary (Resolved Modular Interval and Lorentz Covariance)**. The cutoff $\delta \equiv M_{s}^{-1}$ is placed at fixed proper distance from the causal-diamond waist. Because the diamond is defined by light rays, this local placement introduces no preferred global frame. With local Rindler scaling t≃δτ, the unresolved neighborhoods of the modular tips are excised, giving τ∈[ε,2π−ε]. The canonical screen placement sets ε=1 in modular units. Nearby choices of ε rescale only the universal modular-window factor; the normalized coupling ratios remain fixed by the integer boundary-current levels. The discrete transport layer is confined to the boundary completion near the excised tips. Below $M_{s}$, the resolved interior is described by continuum fields on a smooth manifold; the leading EFT is locally Lorentz invariant, with regulator artifacts appearing only through irrelevant operators suppressed by powers of $E/M_{s}$.

**Corollary (Minimal Regge-Transport Construction)**. Near the spacetime tips (p,q), the transverse cross-section falls below the resolution length $L_{s}=\delta$, forcing smooth $L_{2}$-isotropic modular flow to digitize onto the discrete $L_{1}$ transport layer. Here, an equal-weight signed router maps angular convergence onto an octahedral triangulation of the screen. At each of the six octahedral vertices, four equilateral triangular sectors meet; their angle sum is 4π/3, giving a deficit 2π/3 relative to the flat value 2π. The total deficit is therefore 6(2π/3)=4π, matching the Gaussian-curvature integral of the closed waist $S^{2}$ (equivalently 8π in scalar-curvature convention).

**Corollary (Tip Defect Parameter κ)**. Routing continuous modular flow through the sub-resolution tips incurs an irreducible $L_{2}\to L_{1}$ digitization overhead, encoded by the dimensionless boundary impedance κ. By P5, the resolved waist is a closed finite $S^{2}$ transport graph. Euler closure requires a net positive coordination deficit. In the selected minimal inversion-symmetric sector, this closure is realized by the octahedral router with three antipodal transport-axis pairs, {±x,±y,±z}. At each of the six octahedral poles, four equilateral triangular sectors meet. The Regge angular deficit is 2π−4(π/3)=2π/3, so the normalized local defect fraction is (2π/3)/(2π)=1/3. The six poles sum to 4π, giving the Gaussian-curvature closure of the sphere. Operationally, when a minimal unresolved update reaches a tip, isotropy forbids assigning it to a preferred transport axis. Its scalar effect is therefore distributed equally over the three independent axes, giving the spatial factor 1/3. The remaining normalization comes from the intrinsic modular clock; the modular automorphism of the boundary algebra generates a KMS orbit with Euclidean period 2π. Thus, the per-tip leakage is the one-axis defect fraction averaged over one complete modular cycle: κ=(1/3)(1/(2π))=1/(6π). A full causal-diamond modular cycle crosses both tips *p* and *q*, so the cycle-integrated leakage is 2κ.

**Corollary (Transport-Orientation Redundancy |Γ|)**. The octahedral router contains three antipodal axis pairs, {±x,±y,±z}. Independent reversal of each orientation label generates $\Gamma ≃{\left(Z_{2}\right)}^{3}$; hence, |Γ|=8. These reversals act as equivalent sign conventions rather than propagating degrees of freedom. Under open modular evolution, they coarse-grain into commuting subalgebras (Section 2).

**Corollary (Pixel)**. At finite resolution, a pixel represents a single resolvable algebraic patch on the boundary, defined by the triple (A,H,D) and represented by a node in the mesh. The local algebra A acts on the internal fiber H, while the transport operator *D* provides the nearest-neighbor connectivity routing data between adjacent nodes.

**Corollary (Quantized Holographic Capacity)**. Octahedral transport admits compatible uniform subdivision by edge bisection. After *n* bisections, the number of resolved boundary patches scales as $V_{n}=2+4^{n+1}$. The leading exponential growth is two-dimensional and area-like, while the invariant +2 term continuously records the Euler closure of the sphere. This realizes the boundary as a holographic area-scaling structure.

The axioms operate hierarchically. Axioms P1–P3 define the subsystem: a causal diamond completed by boundary data on a closed $S^{2}$ waist. Axioms P4–P5 supply the intrinsic modular clock and finite physical bandwidth. Axioms P6–P7 establish the minimal topological currents and isotropic transport required for gauge and spinorial data. Once the minimal sector is specified (one-tick resolution, octahedral transport, $Z_{2}$ spin-twist, canonical normalizations) the subsequent structures, including $S_{\mathrm{vac}}$, *N*, κ, $N_{\mathrm{eff}}$, the Hessian blocks and the matching-scale EFT coefficients, arise as constrained outputs (rather than free continuous parameters).

### 1.2. Boundary Architecture, Resolution and Connectivity

A causal diamond O(p,q) (P2) is bounded by two null hypersurfaces (future- and past-directed light-sheets) generated by null rays from *p* and *q* [2]. Their intersection defines a distinguished spacelike waist 2-sphere $S^{2}$, the maximal-area cross-section of the diamond. This waist serves as the local Rindler cut for the small-diamond description: it is the canonical interface on which a local subregion algebra can be completed when $H_{\mathrm{phys}}$ does not factorize (P1–P3) [11,13]. This construction is purely local and does not rely on AdS asymptotics or an AdS/CFT embedding.

Finite resolution (P5) pixelizes the diamond waist: $S^{2}$ operates as a finite set of distinguishable boundary patches (pixels) at resolution δ; sub-δ structures are operationally aliased [19]. Finite resolution is implemented by replacing the idealized null interface, in the local Rindler neighborhood of the waist, with a stretched screen placed at fixed proper distance δ from the cut [25]. This screen is part of the definition of $A_{O}$: it fixes which edge observables are operationally available and, hence, fixes the common operator content on which both $\rho_{O}$ and the reference family $\sigma_{O}\left[\Lambda \right]$ are defined (P3).

Operationally, the null generators of the diamond act as update channels: the screen registers traversing excitations as discrete arrival events, supplying the raw record compared against $\sigma_{O}\left[\Lambda \right]$. The octahedral mesh on the $S^{2}$ waist represents this boundary sector, not a discretized bulk spacetime. The system is defined by graph adjacency, local transport rules and internal node labels.

In the local Rindler regime (P4), the local proper time at the screen scales as t≃δτ [5]. Identifying the minimal resolvable proper time with the screen resolution ($t_{\min}∼\delta$) defines the canonical matching protocol $\varepsilon \equiv \tau_{\min}=1$. Nearby choices of ε rescale the kinematic integration window uniformly across all gauge sectors. Thus the absolute normalization is tied to the canonical screen placement, while the integer-level ratios of inverse couplings remain topologically protected and depend only on the levels $k_{i}$.

Transport across pixels follows the minimal router (P7), implemented in local orthonormal frames of the three-dimensional spatial slice and represented patchwise on the $S^{2}$ waist. A global tangent-frame description on $S^{2}$ is obstructed, so the screen description is local [26,27]. The induced $L_{1}$ step metric approximates the smooth $L_{2}$ isotropic limit; the mismatch becomes relevant near the diamond tips where the cross-section becomes sub-resolution, producing an irreducible digitization overhead, parametrized by κ [28].

The interface is the waist $S^{2}$. Boundary completion represents interior conserved charge sectors as boundary flux labels (P1, P3, P6), reflecting the Gauss-law requirement that charges in a region be encoded by flux through its boundary. The normalization of the local vector response is therefore tied to the surface geometry. We use the standard infrared convention $\alpha \equiv g^{2}/4\pi$. In the present boundary formulation, this convention aligns the gauge coupling with the flux normalization through the closed $S^{2}$ interface.

### 1.3. Topological Budget: Effective Internal Degeneracy

The diamond waist $S^{2}$ serves as the observer’s operational horizon: an instantaneous coherency screen formed by the unique spatial intersection where the past light cone (incoming data from initiation *p*) meets the future light cone (outgoing responses registered at *q*). To determine the effective internal degeneracy *N* of this screen, we define the baseline vacuum capacity functional $S_{\mathrm{vac}}\equiv \sum_{i}S_{i}$ from the topological, tip and spin structures specified by P2–P7. This functional counts internal response depth per resolved boundary patch, not the number of patches. The 2π modular periodicity gives this functional its KMS interpretation, while finite resolution makes it a capacity measure for the boundary completion. Physical area enters separately through the number of surface patches ($A/L_{s}^{2}$). Retaining only dimensionless, scale-free terms, $S_{\mathrm{vac}}$ separates macroscopic area from internal edge capacity.

Bulk Topology ($S_{\mathrm{bulk}}$)

Boundary completion requires a topologically closed interface to satisfy the Gauss-law for interior charge encoding. On the simply connected waist $S^{2}$, the available additive, scale-free local curvature integral is the Euler term. We use the scalar-curvature convention, so the Gauss–Bonnet integral gives 8π rather than the Gaussian-curvature value 4π.

The Gauss–Bonnet theorem [29,30] supplies the unique dimensionless closed-waist curvature invariant entering the capacity functional. In the minimal sector, we denote its logarithmic capacity contribution by:

$$S_{\mathrm{bulk}}\equiv \oint_{S^{2}}R^{\left(2\right)}\;dA=4\pi \chi \left(S^{2}\right)=8\pi$$

Here $S_{\mathrm{bulk}}$ is not a thermodynamic entropy and the Gauss–Bonnet theorem does not by itself count microscopic states. It is a dimensionless logarithmic capacity contribution assigned by the finite-resolution boundary architecture. More generally, one could write $S_{\mathrm{bulk}}=\eta \oint_{S^{2}}R^{\left(2\right)}dA$. The selected minimal sector uses the no-extra-parameter normalization η=1, giving $S_{\mathrm{bulk}}=8\pi$. Choosing η≠1 would introduce an additional boundary-capacity parameter and define a different matching sector.

Finite representatives must preserve this closed topology (V−E+F=2). Open geometries are excluded to avoid unconstrained boundary-circle data.

By Gauss–Bonnet, the waist radius drops out: curvature and area scale inversely, leaving only the Euler invariant. The 8π term is therefore not an area entropy, but the baseline information capacity of the closed surface. It acts as a fixed topological overhead for boundary charges and edge modes, similar to topological entanglement entropy [11,13,31,32]. This scale-independent structure ensures the algebraic gluing required by non-factorization and boundary completion (P1–P3).

A hemisphere is excluded in the selected sector because its boundary circle would require additional edge data and would no longer represent the closed Gauss-law interface used for the capacity functional. Geometric, scale-dependent or multiply bounded choices define different capacity functionals outside the chosen architecture.

The transport input for the capacity budget is the octahedral router (z=6) (P7). A closed modular cycle must treat each local direction and its reverse symmetrically; otherwise, the transport layer would introduce directional bias. Tetrahedral coordination (z=4) lacks antipodal pairs and fails inversion symmetry, while an icosahedral router (z=12) adds transport directions beyond those needed to span three dimensions. Within the signed, inversion-symmetric router class, the minimal spanning set is {±x,±y,±z}. Thus z=6 is the minimal transport architecture used in the capacity count.

Tip Defect Parameter (κ)

At the diamond tips (p,q), the transverse cross-section falls below the resolution length $L_{s}\equiv \delta$ (P5). Smooth modular flow can no longer be represented as a continuous transverse field there. It must be recorded as a discrete boundary update on the $L_{1}$ transport layer selected by P7.

Physically, the tip is the place where a smooth isotropic flow is forced through a finite, graph-local router. The minimal inversion-symmetric router has three antipodal transport-axis pairs, {±x,±y,±z}. Since no axis is preferred, one unresolved tip update must be distributed equally over the three independent axes, yielding the spatial factor 1/3. The same factor appears geometrically in the octahedral Regge construction. A closed triangular transport mesh cannot form a sphere without positive curvature defects. In the octahedral seed graph, four equilateral triangular sectors meet at each pole, so the angular deficit is 2π−4(π/3)=2π/3. The normalized missing angular fraction is, therefore, (2π/3)/(2π)=1/3. The six poles sum to 4π, ensuring the sphere curvature closure.

The remaining normalization comes from the intrinsic modular clock; the modular automorphism of the boundary algebra generates a KMS orbit with Euclidean period 2π. Expressing the per-tip defect as a leakage rate over one full modular cycle gives:

$$\kappa =\frac{1}{3\times 2\pi }=\frac{1}{6\pi }.$$

A complete causal-diamond modular cycle crosses both tips *p* and *q*, so the cycle-integrated leakage is 2κ. This is not an arbitrary normalization. It is the finite-resolution impedance of the diamond tips: the cost of routing smooth modular flow through a closed, isotropic, discrete boundary graph.

The same tip impedance controls coherent tensor participation below and the open-system leakage developed in Section 2.

Twist Contribution ($S_{\mathrm{twist}}$)

The discrete transport structure (P7) must close under frame transport. This requires lifting local rotations from SO(3) to SU(2), thereby supporting local spinors. On the boundary algebra, this lift appears as a $Z_{2}$ grading on closed modular loops.

The contribution counted here is not the Hilbert-space dimension of a local spin-1/2 fiber, which belongs to the matter transport sector. The capacity term instead counts the minimal topological wiring required for the boundary algebra to support the spin-parity holonomy associated with this $Z_{2}$ grading. Intuitively, the spinor and the twist count different things. The spinor is the object being transported; the twist is the boundary gluing rule that makes this transport globally consistent.

In the selected minimal non-Abelian edge sector, this wiring is represented by an Ising/Majorana twist defect σ [33], whose fusion rule σ×σ=1+ψ implies $d_{\sigma }^{2}=2$ and therefore $d_{\sigma }=\sqrt{2}$. Physically, two such boundary twists fuse into an ordinary binary fermion-parity channel. A single twist therefore contributes an irreducible half-bit to the logarithmic capacity budget: $S_{\mathrm{twist}}=\ln d_{\sigma }=\ln\sqrt{2}$.

Choosing $d_{\sigma }=2$ would count the local spinor representation rather than the boundary topological twist, thereby double-counting spinorial degrees of freedom already carried by the local matter sector. The $\sqrt{2}$ contribution supplies only the minimal boundary spin-parity grading required to close the finite-resolution algebra; it does not introduce a propagating bulk anyon in 3+1 dimensions.

In summary, the capacity contribution follows this topological hierarchy:

$$SO\left(3\right)\to SU\left(2\right)\Rightarrow Z_{2}\;\mathrm{grading}\Rightarrow \mathrm{twist}\;\mathrm{defect}\Rightarrow d_{\sigma }=\sqrt{2}\Rightarrow S_{\mathrm{twist}}=\ln\sqrt{2}.$$

Resulting Effective Internal Degeneracy (*N*)

These three contributions arise from independent requirements: closed algebraic gluing, modular-cycle closure and spinorial lift. In this product configuration, their associated state spaces factorize, so their logarithmic capacities add, yielding the total vacuum capacity per pixel:

$$S_{\mathrm{vac}}=8\pi +\frac{1}{6\pi }+\ln\sqrt{2}.$$

Because $S_{\mathrm{vac}}$ is dimensionless and additive, it has the form of a logarithmic capacity. As in statistical mechanics and holographic entropy, where S=lnΩ encodes effective state multiplicity, exponentiation converts this capacity budget into a multiplicative response capacity [16,34,35]. We therefore define the effective channel multiplicity of the selected boundary sector by $N\equiv e^{S_{\mathrm{vac}}}$:

$$\begin{array}{c} N=\sqrt{2}\;\exp\;\left(8\pi +\frac{1}{6\pi }\right)\approx 1.23\times 10^{11} \end{array}.$$

As an exponentiated effective capacity rather than a microscopic Hilbert-space dimension, *N* need not be an integer. It quantifies the intensive internal response capacity assigned to each patch on the boundary algebra. Confined strictly to the boundary completion, this multiplicity introduces no additional propagating bulk fields.

### 1.4. Constitutive Relation (Coherent Participation)

With the total internal capacity *N* fixed by the boundary architecture, gravitational stiffness arises only from the phase-coherent tensor subset that survives a full modular cycle. Channels that lose their quantum phase at the geometric tips thermalize into entropy, leaving only the protected fraction to sustain macroscopic geometric stress.

This coherent participation fraction is fixed below by the tip impedance κ, allowing us to calibrate the resolution scale $M_{s}$ using the measured Newton’s constant. Extensive scaling follows naturally from thermodynamic and holographic constraints (P4, P5). We use the reduced Planck mass $M_{P}$ throughout, so that the Einstein–Hilbert term is normalized as $(M_{P}^{2}/2)R$.

Let $\Pi_{\mathrm{coh}}$ denote the modular-cycle coherent projector on the resolved boundary channel space. The elementary tip impedance fixes the normalized coherent fraction:

$$\frac{N_{\mathrm{eff}}}{N}=\frac{\mathrm{Tr}\Pi_{\mathrm{coh}}}{\mathrm{Tr}1}=\kappa ,\;N_{\mathrm{eff}}\equiv \kappa N.$$

Equivalently, Tr1=N and $\mathrm{Tr}\Pi_{\mathrm{coh}}=N_{\mathrm{eff}}$.

Geometrically, the causal-diamond tips manifest as the fundamental defects of the resolved region (P5). This assigns κ two algebraic roles: an additive capacity weight in the logarithmic budget and a multiplicative coherent participation fraction in the tensor response. For both, κ is structurally fixed by clock and transport normalizations (P4, P7).

*N* represents a vast set of parallel quantum information channels available to the tensor response. In the linear regime, coherent channels decouple quadratically, so their effective stiffnesses add in the macroscopic Hessian [36,37]. Normalizing each active channel by the substrate scale $M_{s}^{2}$ gives the macroscopic tensor stiffness:

$$M_{P}^{2}=N_{\mathrm{eff}}M_{s}^{2}.$$

This relation is a dimensional calibration, not a prediction of Newton’s constant. The boundary architecture first determines the dimensionless coherent stiffness depth $N_{\mathrm{eff}}$. The observed value of *G* is then used exactly once to assign the physical scale $M_{s}$, setting the units, while $N_{\mathrm{eff}}$ and the emergent tensor structure are fixed independently. This logic is therefore structurally non-circular: dimensionless observables, such as tensor-response sum rules, probe the emergent structure directly rather than rederiving *G*.

Within this calibrated description, $M_{P}$ is not introduced as an independent microscopic resolution scale, but rather as the observed macroscopic stiffness assigned to the coherent effective channel capacity. This stiffness additivity aligns with spectral and induced-gravity viewpoints, where effective couplings are controlled by mode counting, and with species-bound logic, where gravitational strength is diluted by the number of active degrees of freedom [37,38].

Within the selected minimal sector, the numerical structure is rigid: the closed-waist capacity weight, the tip impedance, the spin-twist sector, the one-tick modular resolution, and the signed three-axis transport architecture fix *N* and $N_{\mathrm{eff}}=\kappa N$. These inputs are not continuous parameters. Changing any of them shifts the theory to a different boundary sector or matching convention.

### 1.5. Numerical Calibration

We now calibrate the resolution scale $M_{s}$ using the measured Newton’s constant, expressed through the reduced Planck mass $M_{P}\equiv {\left(8\pi G\right)}^{-1/2}\approx 2.435\times 10^{18}\;\mathrm{GeV}$. With the capacity functional above, $N_{\mathrm{eff}}=\kappa N≃6.51\times 10^{9}$. Solving $M_{P}^{2}=N_{\mathrm{eff}}M_{s}^{2}$ for the resolution scale gives:

$$\begin{array}{c} M_{s}=\frac{M_{P}}{\sqrt{N_{\mathrm{eff}}}}\approx 3.02\times 10^{13}\;\mathrm{GeV} \end{array}.$$

$M_{s}$ represents the physical resolution limit of the algebra (sharp focus) where the EFT problem is well-posed: gravitational stiffness, gauge couplings and mass gaps enter as matching data, and in quantized sectors some conditions reduce to closed-form constraints (Section 3). This calibrated matching scale lies in the intermediate range often associated with high-scale seesaw models and plateau-inflation phenomenology [39,40].

In reduced-Planck units, the inverse stiffness scale is $M_{P}^{-1}=L_{s}/\sqrt{N_{\mathrm{eff}}}$. Thus $M_{s}$ acts as the fundamental resolution limit, while $M_{P}$ emerges strictly as the collective tensor stiffness of the boundary, eliminating the singular $L_{P}\to 0$ continuum artifact.

Pixel Saturation Limit

Finite resolution (P5) bounds the local excitation capacity of a single boundary pixel. The internal multiplicity *N* includes the spin-twist factor $d_{\sigma }=\sqrt{2}$, which supplies the minimal $Z_{2}$ spin-parity grading. Because this edge twist is not an addressable scalar channel, the saturation estimate uses the non-twist channel count $n_{\mathrm{ch}}\equiv N/\sqrt{2}$.

With $M_{s}$ fixed by the Newton calibration, the single-pixel activation scale is $E_{\mathrm{pix}}\equiv M_{s}/n_{\mathrm{ch}}$. More generally, a local scalar operator with *q* fermionic legs is governed by a single-pixel saturation cap $m_{\max}^{\left(q\right)}=E_{\mathrm{pix}}/q$. Mass deformations on the boundary-completed algebra must be closed gauge-invariant scalar operators. A hypothetical single-leg insertion (q=1) is therefore inadmissible. Higher-valence scalar operators (q≥4) further lower the cap, corresponding to higher-dimensional EFT deformations. The leading admissible mass deformation is instead the gauge-invariant bilinear $\bar{\psi }\psi$, fixing q=2:

$$m_{\max}^{\left(q\right)}=\frac{E_{\mathrm{pix}}}{q}\;\overset{\;\bar{\psi }\psi ,\;q=2\;}{\to }\;m_{\max}^{\left(2\right)}=\frac{E_{\mathrm{pix}}}{2}\approx 174\;\mathrm{GeV}.$$

The resulting upper bound lies close to the observed top-quark mass ($m_{t}\approx 173\;\mathrm{GeV}$). This proximity serves as a heuristic consistency check of the single-pixel saturation cap, not a mass prediction.

Consistency Check: Area Law

We verify that this calibration preserves the Bekenstein–Hawking entropy structure [34,41,42]. For a horizon of area *A*, the standard entropy (in natural units) is $S_{\mathrm{BH}}=A/4G$. Substituting $1/G=8\pi M_{P}^{2}$, we obtain:

$$S_{\mathrm{BH}}=2\pi M_{P}^{2}A=2\pi \left(N_{\mathrm{eff}}M_{s}^{2}\right)A=2\pi N_{\mathrm{surf}}N_{\mathrm{eff}},$$

where $N_{\mathrm{surf}}\equiv A/L_{s}^{2}=AM_{s}^{2}$ counts the resolved boundary pixels on a waist of area *A*. The entropy therefore factors into a geometric pixel count and a coherent internal depth. The two quantities play different roles: $S_{\mathrm{vac}}$ is the per-pixel logarithmic capacity defining $N=e^{S_{\mathrm{vac}}}$, whereas $S_{\mathrm{BH}}$ is the area-extensive entropy obtained by summing the effective tensor-response depth $N_{\mathrm{eff}}=\kappa N$ over all pixels.

Converting to bits (S/ln2) and taking $A_{\cos m}∼4\pi H_{0}^{-2}$ gives $∼10^{122}$ bits. This reproduces the standard Bekenstein–Hawking area law and the familiar cosmic entropy scale, providing a consistency check of the calibration.

The result follows algebraically from the constitutive relation $M_{P}^{2}=N_{\mathrm{eff}}M_{s}^{2}$. It shows that the standard horizon entropy can be represented as a resolved pixel count multiplied by an effective tensor-response depth. Channel multiplicity supplies the required information density without shrinking the pixel size to $L_{P}$, recovering the Bekenstein–Hawking scaling natively at the finite resolution scale $L_{s}$. Within this framework, $M_{s}$ is the physical regulator, while the reduced Planck scale is the collective stiffness scale.

### 1.6. Emergent Time

In a background-independent framework, time is not a global parameter but emerges relationally [3,43]. Inside any single causal diamond, the local quantum state supplies an intrinsic operational clock τ via modular flow [4,5], ordering comparisons within a fixed boundary algebra. Time is strictly diamond-local: one operational tick corresponds to a single distinguishable boundary update at $M_{s}$.

The continuous proper time t≃δτ of a macroscopic observer emerges only after coarse-graining these updates. With the local clock and boundary algebra fixed, diamond dynamics reduces to statistical inference on constant operator content [44,45]. This variational problem matches a finite boundary record to a reference state, rather than evolving in external time.

Physical evolution arises as the resolved domain advances to the next overlapping diamond, where finite-resolution coarse-graining yields local irreversibility. This entropic view of quantum evolution directly motivates the relative-entropy variational principle developed in Section 2.

## 2. Relative-Entropy Functional and Dynamics

Dynamics on a causal diamond emerge not as the time-evolution of a global Wheeler–DeWitt state [1], but as local statistical inference. In algebraic quantum field theory (AQFT), infinite local degrees of freedom (Type III algebras) break standard density matrices and traces. We restore mathematical rigor by evaluating them at finite resolution on the boundary-completed effective algebra $A_{O}$, where the reference KMS state is faithful [18,46]. On this shared algebra, the reduced boundary state $\rho_{O}$ supplies the local data, while geometry and other background fields enter exclusively through a semiclassical reference family $\sigma_{O}\left[\Lambda \right]$. The resolved algebra defines the operational arena: it fixes which physical distinctions the causal diamond can actually register.

While standard entanglement-equilibrium methods rely on continuum locality [6,7,8,47,48,49,50,51,52], this framework anchors the subsystem at finite resolution on a boundary-completed algebra, transforming the variational problem into a matching-scale effective theory at $M_{s}=\delta^{-1}$ [53]. The local dynamics are organized by the relative entropic mismatch functional $I\left[\Lambda ;O\right]=S_{\mathrm{rel}}\left(\rho_{O}∥\sigma_{O}\left[\Lambda \right]\right)$ at fixed $\left(O,A_{O},\delta \right)$ [44,46,54]. Relative entropy therefore measures the information cost of explaining the fixed boundary data with a deformed background Λ. Around a matched reference point, its Hessian naturally decouples into independent tensor, vector and scalar blocks, making a joint matching of all three sectors well-posed.

We keep four inputs separate: the axioms P1–P7, the calibration in Section 1, the selected minimal implementation, and the EFT matching approximations.

We use *N* for total internal channel multiplicity per pixel (boundary patch), $N_{\mathrm{eff}}=\kappa N$ for effective coherent participation, κ for the tip impedance factor, k∈Z for the WZW level, α for the gauge coupling and $M_{s}$ for the resolution scale.

### 2.1. Entropic Variational Principle and Operational Domain

We fix the container $\left(O,A_{O},\delta \right)$ with proper-distance regulator $\delta =M_{s}^{-1}$ ($\delta \ll \ell_{O}\ll R_{\mathrm{curv}}$), varying only the reference family $\sigma_{O}\left[\Lambda \right]$ on the fixed algebra $A_{O}$. We define the variational functional [54]:

$$I\left[\Lambda ;O\right]\;:=\;S_{\mathrm{rel}}\left(\rho_{O}\;∥\;\sigma_{O}\left[\Lambda \right]\right).$$

Boundary completion (P3) supplies the common operator domain on which $S_{\mathrm{rel}}\geq 0$ is well defined and monotone under further restriction [18,46,54,55]. On this fixed algebra, we adopt an exponential reference family as the maximum-entropy formulation compatible with the chosen sources Λ [44,46]:

$$\sigma_{O}\left[\Lambda \right]=\frac{e^{-K_{O}\left[\Lambda \right]}}{\mathrm{Tr}(e^{-K_{O}\left[\Lambda \right]})}.$$

The modular generator $K_{O}\left[\Lambda \right]$ couples the local background multiplet Λ to the boundary operator multiplet O, defining deformations via the inner product:

$$\delta K=\int d^{4}x\;\sqrt{-g}\;\delta \Lambda \left(x\right)\cdot O\left(x\right).$$

This integral serves as the quasi-local continuum shorthand for boundary-smeared operators after modular averaging. In the KMS regime, relative entropy takes the modular free-energy form [54]. At the matched point $\rho_{O}=\sigma_{O}\left[\Lambda_{0}\right]$, reference variations are tangent to a normalized exponential family. The linear relative-entropy term vanishes, reflecting the local entanglement first law [54,56,57]:

$$S_{\mathrm{rel}}\left(\rho ∥\sigma \right)=\Delta \langle K_{\sigma }\rangle -\Delta S\;\left(K_{\sigma }=-\log\sigma \right),\;\delta I{|}_{\Lambda_{0}}=0.$$

With the linear term absent, the restoring response in the local stationary expansion is strictly quadratic, governed by the Hessian.

We vary the reference background $g\mapsto \sigma_{O}\left[g\right]$ at fixed $\left(O,A_{O},\delta \right)$. Metric variations deform $K_{O}\left[g\right]$ and the correlators on the same boundary-completed algebra; they do not move the endpoints (p,q) or the regulator. The regulator remains at fixed proper distance $\delta =M_{s}^{-1}$ from the waist, so the unresolved tip region stays in the same operational class and carries the tip parameter κ.

### 2.2. Hessian Bridge, Spectral Representation and EFT Coefficients

Local background fields (metric $g_{\mu \nu }$, gauge field $A_{\mu }$, scalar φ) couple on the boundary algebra to three composite operators: the stress tensor $T^{\mu \nu }$, the conserved current $J^{\mu }$, and the scalar density *M*. Within the finite-resolution boundary-completed algebra $A_{O}$, the leading Hessian kernel $G_{IJ}\left(x,y\right)$ block-diagonalizes across these source sectors at the symmetric matched KMS reference. This separation follows from the source labels preserved by the boundary architecture.

Modular flow, generated by the geometric boost $K_{\mathrm{mod}}$ (P4), is fixed by the causal diamond and its waist. The source-label symmetries retained by the boundary-completion architecture commute with the reference modular flow, $[K_{\mathrm{mod}},Q]=0$ [11,58]. Metric, gauge-connection, and scalar deformations therefore transform as inequivalent source modules. Any nonzero mixed Hessian block must define a symmetry-preserving map between inequivalent source modules.

Schur’s lemma, together with Ward identities, forces such maps to vanish in the leading quadratic response: $G_{IJ}\left(x,y\right)=0$ for I≠J.

The discrete $L_{1}$ router (P7) contributes only subleading anisotropic corrections that are assigned to the $O(M_{s}^{-2})$ EFT remainder and average to zero over the 2π modular period under octahedral projection. The $Z_{2}$ twist is a topological superselection rule, partitioning the algebra into bosonic and fermionic sectors whose center is preserved by modular flow [43,58]. At the symmetric reference, no mixed correlators survive the KMS-weighted projection at leading order. To fix normalizations, the source-operator pairing is

$$\delta K=\int d^{4}x\sqrt{-g}\left(\frac{1}{2}\;\delta g_{\mu \nu }\;T^{\mu \nu }+\delta A_{\mu }\;J^{\mu }+\delta \varphi \;M\right),$$

where the factor 1/2 is the usual metric-source convention.

These sources probe three distinct mismatches: geometric stress, charge transport and local occupancy. Expanding around the reference KMS state $\Lambda_{0}$ gives the standard variational series:

$$I\left[\Lambda_{0}+\delta \Lambda \right]=I\left[\Lambda_{0}\right]+\int d^{4}x\;\delta \Lambda \left(x\right)\cdot {\left.\frac{\delta I}{\delta \Lambda (x)}\right|}_{\Lambda_{0}}+\frac{1}{2}\;\int \int \;d^{4}x\;d^{4}y\;\delta \Lambda \left(x\right)\cdot \underset{G(x,y)}{\underset{︸}{{\left.\frac{\delta^{2}I}{\delta \Lambda (x)\delta \Lambda (y)}\right|}_{\Lambda_{0}}}}\cdot \delta \Lambda \left(y\right)+{O(∥\delta \Lambda ∥}^{3})$$

At equilibrium, $\rho_{O}=\sigma_{0}$, so $I[\Lambda_{0}]$ and the first variation vanish. The leading response is therefore quadratic and governed by the Hessian kernel:

$$G\left(x,y\right):={\left.\frac{\delta^{2}I}{\delta \Lambda (x)\delta \Lambda (y)}\right|}_{\Lambda_{0}}.$$

Centering tangent modular operators, the Hessian becomes the connected Kubo–Mori correlator, the KMS-weighted two-point response at the matched reference state (P4) [54,59,60]:

$${\langle \delta K|\delta K\rangle }_{\sigma }=\int_{0}^{1}ds\;\mathrm{Tr}\left(\sigma^{s}\delta K\;\sigma^{1-s}\delta K\right).$$

The quadratic contribution to *I* is, therefore, $\frac{1}{2}{\langle \delta K|\delta K\rangle }_{\mathrm{KM}}$ near the matched KMS state. The Hessian is the local stiffness matrix of distinguishability [56].

Because modular flow acts as a geometric boost (P4) and transport-layer anisotropy averages to zero over a full 2π orbit, the reference vacuum is isotropic and parity-even. Local deformations, therefore, decompose into orthogonal spin channels (transverse-traceless tensors, conserved vectors, scalars), with the $Z_{2}$ grading (P7) isolating the fermionic sector. By symmetry, Kubo–-Mori cross-pairings between these representations vanish:

$${\langle \delta g|\delta A\rangle }_{\mathrm{KM}}={\langle \delta g|\delta \varphi \rangle }_{\mathrm{KM}}={\langle \delta A|\delta \varphi \rangle }_{\mathrm{KM}}=0.$$

This block decomposition applies to the leading quadratic Kubo–Mori response at the symmetric matched KMS reference state. It is a tangent-space statement. It does not exclude higher-order or nonlinear mixed interactions. KMS compatibility alone is not sufficient for an operator to enter the leading Hessian. The operator must also satisfy the full source-label constraints: gauge invariance, index contraction, Ward identities, parity, isotropy and modular labels.

The Hessian therefore block-diagonalizes at leading order (mixed terms either vanish under projection or enter at higher order).

**Figure 1:**
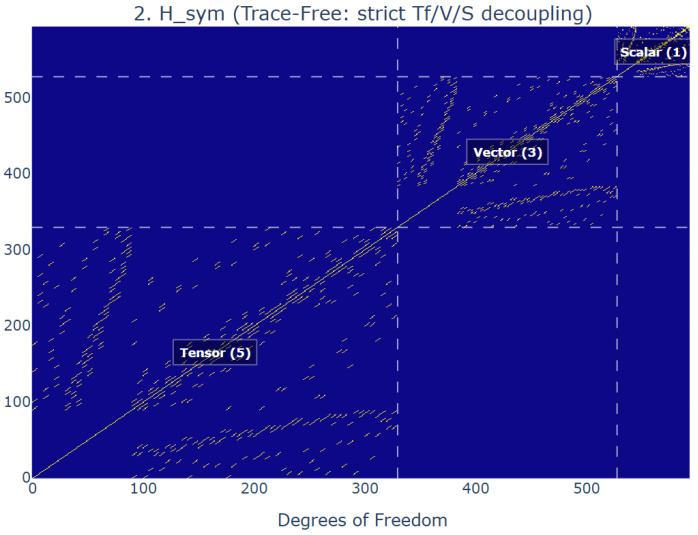
Symmetry-projected finite-boundary Hessian. Simulated via the finite-lattice code [61], the octahedral projection separates the trace-free tensor, vector and scalar source modules into clean orthogonal response blocks. All off-block entries vanish to numerical precision.

This theoretical block-diagonalization is verified computationally in the supplementary toolbox hessian_sector_decoupling.py [61]. The simulation builds an octahedrally refined finite $S^{2}$ boundary, starts from a generic dense local fiber Hessian with tensor/vector/scalar mixing, and then applies the finite octahedral Reynolds projection. The projected heatmap in Figure 1 shows the resulting trace-free tensor, vector and scalar response blocks. The dense pre-projection matrix and the contamination controls are provided in the dashboard generated by the companion code [61].

KMS compatibility alone does not place a mixed-gradient operator in the leading tangent Hessian. Terms such as $F_{\mu \nu }\partial^{\mu }\phi$ and $T^{\mu \nu }\partial_{\mu }\phi \;\partial_{\nu }\phi$ either require scalar completion, vanish at the homogeneous matched reference where $\partial_{\mu }\phi =0$, or reduce to total derivatives on the closed $S^{3}\times S^{1}$ history manifold. Any remaining mixed-gradient contributions belong to the $M_{s}^{-2}$-suppressed EFT remainder and do not affect the leading tensor/vector/ scalar matching.

Low-momentum sources ($\left|p\right|\ll M_{s}$) effectively commute with the modular background. This kinematic suppression reduces the Kubo–-Mori kernel to its leading gradient expansion, yielding a symmetric spectral trace over the covariant transport operator (P7):

$${\langle \delta K|\delta K\rangle }_{\mathrm{KM}}\approx \mathrm{Tr}\left(\delta K\;f(D_{A}^{2}/M_{s}^{2})\;\delta K\right),$$

where the positive modular window f(τ) is obtained by integrating the KMS kernel over the resolved modular interval [ε,2π−ε] with ε=1.

The same finite resolution $M_{s}\equiv \delta^{-1}$ that defines the stretched horizon also sets the physical EFT cutoff (Nyquist frequency). Deformations with physical momentum $|p|>M_{s}$ are unresolved and integrated out of the effective description (effectively aliased away). Evaluated on the $S^{3}\times S^{1}$ history manifold, the heat-kernel trace extracts the local EFT coefficients. This spectral operation isolates the numerical prefactors while preserving the physical source insertions. Operationally, the heat kernel counts the resolved modes available to carry each response and packages them into local EFT coefficients.

Expressing the window *f* via Laplace transform, we insert the short-time heat-kernel expansion $\mathrm{Tr}\;e^{-tD^{2}}∼\sum_{n}a_{n}t^{(n-4)/2}$ [62,63]. With irrelevant corrections constrained by the Monotone Spectral Suppression corollary (P5), this yields finite EFT coefficients $a_{n}$:

$$\mathrm{Tr}\;f\left(D_{A}^{2}/M_{s}^{2}\right)∼\sum_{n}a_{n}\left(\int_{0}^{\infty }dt\;t^{(n-4)/2}\tilde{f}\left(t\right)\right)M_{s}^{4-n}.$$

Note that the standard Seeley–DeWitt coefficients encode the local geometric invariants ($a_{2}\propto R$ and $a_{4}\propto R^{2},F^{2}$).

At low derivative order, locality and symmetry fix the operator basis, mapping the independent Hessian blocks directly to the physical effective action:

$$\delta^{2}I≃\underset{\Rightarrow \int d^{4}x\sqrt{-g}\;\frac{M_{P}^{2}}{2}R}{\underset{︸}{\frac{1}{2}\;\int \int \;\delta g_{\mu \nu }\;G_{TT}\;\delta g_{\rho \sigma }}}\;+\;\underset{\Rightarrow \int d^{4}x\sqrt{-g}\;\frac{1}{4g^{2}}F^{2}}{\underset{︸}{\frac{1}{2}\;\int \int \;\delta A_{\mu }\;G_{JJ}\;\delta A_{\nu }}}\;+\;\underset{\Rightarrow \int d^{4}x\sqrt{-g}\;L_{\mathrm{mass}}}{\underset{︸}{\frac{1}{2}\;\int \int \;\delta \varphi \;G_{MM}\;\delta \varphi }}.$$

To isolate the resulting dynamic restoring forces, we absorb all static vacuum-energy contributions directly into the reference state. At leading two-derivative order, the tensor block directly matches the Einstein–Hilbert coefficient. We extract these local EFT parameters by evaluating the heat-kernel trace over the modular manifold $S^{3}\times S^{1}$ at the finite bandwidth $M_{s}$. Below this threshold, standard renormalization group running takes over. Consequently, the cutoff $M_{s}$ is not an ad hoc external regulator [64,65], but the physical threshold where the boundary algebra stops distinguishing finer structure.

### 2.3. Coupling Factorization and Spectral–Volume Correspondence

Quasi-locality factorizes the leading quadratic response of slowly varying fields into an effective four-volume, an intensive coupling and a local average. Absorbing the universal density-of-states factors yields [62,63]:

$${\mathrm{Tr}}_{D}1_{A}≃NM_{s}^{4}{\mathrm{Vol}}_{4}\left(D\right)\;\Rightarrow \;{\mathrm{Vol}}_{4}\left(D\right)=\frac{{\mathrm{Tr}}_{D}1_{A}}{NM_{s}^{4}}$$

This acts as a finite-resolution analogue of Weyl’s law. The spectral trace counts the total resolved states; dividing it by $NM_{s}^{4}$ factors out the microscopic capacity and leaves only the coarse-grained four-volume.

Here, *N* counts the available internal response channels. The effective volume is inferred from the spectral mode count rather than assumed as a primitive coordinate measure. The tensor sector samples the coherently participating subset $N_{\mathrm{eff}}=\kappa N$, making Einstein stiffness extensive in channel number. By contrast, the vector and scalar sectors are normalized per channel, so the gauge stiffness $1/g^{2}$ and mass susceptibilities remain intensive responses at $M_{s}$.

The spectral trace over the minimal compact operational history then establishes a spectral–volume correspondence. In the small-diamond/KMS regime, modular time forms a thermal circle $S^{1}$ [4,18]. Identifying the spatial part as two $B^{3}$ halves glued across the waist ($B^{3}\cup_{S^{2}}B^{3}≃S^{3}$), the minimal manifold for the trace is $S^{3}\times S^{1}$ [62].

This correspondence converts the spectral trace into an effective four-volume at resolution $M_{s}$. The trace counts distinguishable response channels, and this factorization separates extensive tensor stiffness from intensive charge susceptibilities.

### 2.4. Tensor Sector: Gravity as Extensive Stiffness

Applying the Hessian mapping to $\delta g_{\mu \nu }$, the tensor block $G_{TT}$ measures the stiffness of the geometric pixel under modular flow. In the low-curvature regime $\left|R\right|\ll M_{s}^{2}$, linear response around the modular vacuum governs the dynamics [6]. In the small-diamond KMS regime, the modular generator couples $\delta g_{\mu \nu }$ to the stress tensor $T^{\mu \nu }$ (via the conformal Killing vector), supplemented by the boundary completion term required on bounded regions. Its variation reproduces the geometric entropy functional of the cut (area/Wald-type) [4,11,16,34].

With the first variation vanishing at equilibrium, the second variation governs the tensor response. At low curvatures, locality, parity and diffeomorphism invariance restrict this leading two-derivative response to the Einstein–Hilbert invariant [7,10,57,66,67]. Absorbing the identity contribution into the reference state isolates gravitational stiffness from the vacuum-energy prescription. The absolute coefficient for this emergent tensor structure is then assigned using the macroscopic Newton calibration ($M_{P}^{2}=N_{\mathrm{eff}}M_{s}^{2}$) imported from Section 1. Gravity is extensive because coherent boundary channels resist geometric deformation collectively.

### 2.5. Vector Sector: Gauge as Intrinsic Susceptibility

Applying the Hessian mapping to $\delta A_{\mu }$, the vector block $G_{JJ}$ measures the intrinsic susceptibility of the boundary current, specifically the linear response of the boundary state to an external connection. A weak background connection couples to the conserved edge current $J^{\mu }$, required by the CS/WZW structure [21,24], through the modular generator:

$$\delta K\left[A\right]=\int d^{4}x\sqrt{-g}\;A_{\mu }\left(x\right)\;J^{\mu }\left(x\right).$$

Ward identities enforce gauge invariance, restricting this vector response to the field strength tensor. Because the reference state is parity-even, the matching isolates the standard kinetic term ($F_{\mu \nu }F^{\mu \nu }$), leaving parity-odd topological terms unconstrained. Gauge susceptibility emerges as a geometric property determined by the fluctuation-dissipation relation of the boundary algebra [59,60].

By P5 corollary, finite resolution truncates the operational modular interval to τ∈[1,2π−1]. On the 1D modular orbits, the angularly averaged chiral current takes the standard affine level form $\langle J\left(\tau \right)J\left(0\right)\rangle =\frac{k}{4}\sin^{-2}\left(\tau /2\right)$ [68]. The level k∈Z is defined in the trace convention fixed by the boundary current normalization.

Let ϵ be a formal symmetric endpoint for the modular window. The raw modular current fluctuation is:

$$\chi_{\mathrm{pix}}\left(\epsilon \right)=\frac{k}{4}\int_{\epsilon }^{2\pi -\epsilon }\csc^{2}\left(\tau /2\right)\;d\tau =k\cot\left(\epsilon /2\right).$$

With a sensitivity of $d\ln\chi_{\mathrm{pix}}/d\epsilon =-1/\sin\epsilon$, this raw fluctuation clearly depends on the chosen window. However, this dependence is purely kinematic and universal across all current sectors. Factoring out the common modular-window factor, $I_{\mathrm{mod}}\left(\epsilon \right)=4\cot\left(\epsilon /2\right)$, isolates the physical susceptibility:

$${\bar{\chi }}_{\mathrm{pix}}\left(\epsilon \right)=\frac{\chi_{\mathrm{pix}}\left(\epsilon \right)}{I_{\mathrm{mod}}\left(\epsilon \right)}=\frac{k}{4}.$$

The endpoint dependence cancels exactly, leaving only the integer current level *k*. All group-theory factors and trace normalizations are absorbed into this *k*.

The canonical choice ϵ=1 represents one minimal modular update on the discrete boundary algebra. It fixes the readout convention at the matching scale $M_{s}$; it is not a continuously sliding Wilsonian cutoff. Standard renormalization-group running applies only below $M_{s}$.

Physically, ϵ merely scales the kinematic readout, while the true gauge stiffness is fixed by the normalized topological response. Applying the volume-coupling factorization to match this quadratic response to the canonical gauge term $-\frac{1}{4g^{2}}F^{2}$ identifies the inverse coupling $1/g^{2}=4{\bar{\chi }}_{\mathrm{pix}}=k$. Utilizing the standard flux convention $\alpha \equiv g^{2}/4\pi$, this yields the matching-scale inverse coupling:

$$\frac{1}{g^{2}}=4{\bar{\chi }}_{\mathrm{pix}}=k\;\Rightarrow \;\alpha^{-1}\left(M_{s}\right)=4\pi k,\;k\in Z.$$

This matching applies to each normalized current block, with Abelian factors requiring a fixed charge convention. Thus, $\alpha^{-1}\left(M_{s}\right)=4\pi k$ is a rigidly quantized boundary condition, not a continuum cutoff artifact. The continuous low-energy gauge couplings are then obtained by standard renormalization-group evolution from these discrete topological boundary data.

### 2.6. Scalar Sector: Mass as Occupancy Stiffness

Applying the Hessian mapping to δφ, the scalar block $G_{MM}$ measures the susceptibility of the selected scalar density; for fermions, the minimal gauge-invariant density is $M=\bar{\psi }\psi$. We couple the source φ to this selected operator via:

$$\delta K_{M}=\int d^{4}x\sqrt{-g}\;\delta \varphi \left(x\right)\;\bar{\psi }\left(x\right)\psi \left(x\right).$$

In the symmetry decomposition, $\bar{\psi }\psi$ is a Lorentz scalar, so mass resides in this block. The scalar response identifies the mass operator as the leading relevant deformation, serving as a matching prescription (rather than an independent spectrum derivation):

$$S_{\mathrm{mass}}\approx -\int d^{4}x\sqrt{-g}\;m\;\bar{\psi }\psi .$$

Mass therefore emerges as the energetic cost of maintaining a local fermion occupancy. The $Z_{2}$ grading (P7) admits spinor representations [69], whose discrete transport reduces locally to the Dirac operator:

$$D_{\mu }=\partial_{\mu }+\frac{1}{4}\omega_{\mu }^{ab}\gamma_{ab}-iA_{\mu }^{a}T^{a}.$$

Independent of finite capacity, fermionic statistics enforce binary occupancy n∈{0,1}. In discrete modular time, a local mass gap acts as the on-site phase accumulated per update. Parameterizing $\theta_{f}≃m_{f}/E_{\mathrm{pix}}$ (subject to $|\theta_{f}|\leq \pi$) and using the per-pixel energy budget $E_{\mathrm{pix}}$ from Section 1 gives the heuristic scaling rule $m\propto m_{\mathrm{anchor}}/\Omega_{\mathrm{int}}$, where $\Omega_{\mathrm{int}}$ counts the resolved internal modes accessible at $M_{s}$ [62,63].

### 2.7. Unified Matching-Scale Action at $M_{s}$

The operators derived above govern the low-curvature regime. To model finite-resolution saturation, we restrict the matching to the reduced homogeneous scalar-curvature sector. In this sector, the only retained local four-derivative invariant is $R^{2}$; the Gauss–Bonnet density is topological and anisotropic curvature structures are not retained.

Finite resolution (P5) supplies the sole intrinsic scale $M_{s}$, so the saturation completion of Einstein stiffness takes the form $\left(M_{P}^{2}/2\right)R+\lambda R^{2}$, where λ is the dimensionless quadratic coefficient that limits maximum curvature. Extracting λ therefore reduces to comparing the *R* and $R^{2}$ coefficients within a single scalar heat-kernel expansion. Together with the isotropic, inversion-symmetric transport layer (P7), this finite capacity selects the conformal Laplacian as the massless scalar transport operator in the minimal bosonic implementation:

$$\Delta_{c}=-\nabla^{2}+E,\;E=R/6.$$

This conformal choice (P7) eliminates the arbitrary curvature coupling ξ of a generic scalar operator $-\nabla^{2}+\xi R$, isolating the gravitational scalar sector while leaving the spinorial lift of matter to separate fermionic blocks. On the closed $S^{3}\times S^{1}$ history manifold, total derivatives vanish and $\Omega_{\mu \nu }=0$. The matching applies identical modular-moment normalization to the retained curvature terms, defining the selected spectral protocol.

The relevant Seeley–DeWitt coefficients then reduce to local curvature integrals. The two-derivative term is $a_{2}={\left(4\pi \right)}^{-2}\int d^{4}x\;\sqrt{g}\;\left(R/3\right)$. On $S^{3}\times S^{1}$, curvature resides entirely on the constant-curvature $S^{3}$ factor, while the $S^{1}$ factor is flat. Hence $R_{\mu \nu }R^{\mu \nu }=R_{\mu \nu \rho \sigma }R^{\mu \nu \rho \sigma }=R^{2}/3$. Substituting these identities and E=R/6 into the standard four-dimensional $a_{4}$ coefficient [62] cancels the Ricci-squared and Riemann-squared terms, collapsing the surviving scalar polynomial to:

$$a_{4}={\left(4\pi \right)}^{-2}\int d^{4}x\;\sqrt{g}\;\frac{R^{2}}{18}.$$

This directly isolates the scalar-curvature projection of the spectral response. The reduced local curvature coefficients are $c_{R}=1/3$ and $c_{R^{2}}=1/18$, so $c_{R^{2}}/c_{R}=1/6$. Matching the *R* term to the Einstein–Hilbert normalization $(M_{P}^{2}/2)R$ and accounting for the relative mass dimension $M_{s}^{-2}$ fixes the quadratic coefficient λ:

$$\lambda =\frac{M_{P}^{2}}{2}\left(\frac{1}{6M_{s}^{2}}\right)=\frac{M_{P}^{2}}{12M_{s}^{2}}=\frac{N_{\mathrm{eff}}}{12}\approx 5.42\times 10^{8}.$$

Below the matching scale $M_{s}$, Wilsonian decoupling confines finite-resolution corrections to higher-derivative operators. At infrared scales $E\ll M_{s}$, the locked coefficient receives a relative correction of order $O(E^{2}/M_{s}^{2})$. These subleading effects enter through the suppressed EFT remainder, preserving the plateau coefficient as a fixed matching-scale boundary condition without introducing a new free parameter.

Within this reduced homogeneous sector, the operational inputs of finite capacity and isotropic transport have a calculable EFT consequence: the heat-kernel trace fixes the $R^{2}$ saturation coefficient λ by the ratio $M_{P}^{2}/M_{s}^{2}$. This large parameter reflects the hierarchy between the collective stiffness scale $M_{P}$ and the fundamental scale $M_{s}$, yielding $M_{R}=M_{s}$ and placing the high-curvature completion in the Starobinsky/plateau class [40,70].

At the matching scale, the local action is the EFT representative whose second variation reproduces the block-diagonal Kubo–Mori Hessian. Because the response sectors are strictly orthogonal at the symmetric matched KMS reference, the unified action contains no leading mixed interactions. Ward identities and derivative counting map the tensor, vector and scalar/spinorial response blocks to $R+R^{2}$, $F_{\mu \nu }F^{\mu \nu }$ and the Dirac kinetic plus mass-deformation terms. The resulting matching-scale action is:

$$L=\underset{\begin{array}{c} \mathrm{Einstein}\;\mathrm{stiffness}\;+\;R^{2}\;\mathrm{plateau}\\ \mathrm{tensor}\;\sec\mathrm{tor},\;\mathrm{extensive}\;\mathrm{in}\;N_{\mathrm{eff}} \end{array}}{\underset{︸}{\frac{M_{P}^{2}}{2}R+\frac{M_{P}^{2}}{12M_{s}^{2}}R^{2}}}-\underset{\begin{array}{c} \mathrm{Yang}–\mathrm{Mills}\;\mathrm{kinetic}\\ \alpha^{-1}\left(M_{s}\right)=4\pi k,\;\mathrm{vector}\;\sec\mathrm{tor} \end{array}}{\underset{︸}{\frac{1}{4g{\left(M_{s}\right)}^{2}}F_{\mu \nu }F^{\mu \nu }}}+\underset{\begin{array}{c} \mathrm{Dirac}\;\mathrm{kinetic}\;+\;\mathrm{mass}\\ \mathrm{spinorial}/\mathrm{scalar}\;\sec\mathrm{tor} \end{array}}{\underset{︸}{\bar{\psi }i\gamma^{\mu }D_{\mu }\psi -m\bar{\psi }\psi }}+\cdots .$$

Here, $F_{\mu \nu }$ is the Yang–Mills field strength, ψ the Dirac fermion and $D_{\mu }$ the gauge- and spin-covariant derivative. The tensor sector yields the emergent Einstein–Hilbert structure (anchored by the macroscopic $M_{P}$ calibration) and, within the reduced homogeneous curvature sector, fixes its dimensionless $R^{2}$ plateau completion. The vector sector contributes the Yang–Mills kinetic term with $\alpha^{-1}\left(M_{s}\right)=4\pi k$, the spinorial transport block supplies the Dirac kinetic term and the scalar block contributes the leading mass deformation. Within this selected sector, no additional continuous coefficient is introduced beyond the implementation choices and constitutive calibration.

This Lagrangian does not quantize gravity; instead, it matches the leading gravitational, gauge and matter sectors as orthogonal projections of the relative-entropy quadratic response on $A_{O}$ at the matching scale, where the discrete update layer admits a continuum field description. The extracted coefficients therefore characterize the observation layer (the lens) rather than the underlying microscopic object, encoding above-cutoff responses into fixed parameters and preventing them from being reintegrated in the low-energy effective theory. Below this cutoff, standard EFT renormalization applies.

### 2.8. Relation to Continuum Approaches

This formulation reorganizes standard quantum-gravity logic around finite-resolution local inference, complementing continuum entropic programs [6,8,49,71,72] by explicitly enforcing background independence and finite resolution. Rather than deriving classical spacetime from a microscopic path integral, gravitational, gauge, and matter sectors emerge jointly as the structured response of the boundary-completed finite-resolution algebra. Within the causal diamond $\left(O,A_{O},\delta \right)$, non-factorization (P1) forces boundary completion, while finite resolution (P5) supplies the physical bandwidth $M_{s}=\delta^{-1}$. This renders the inference problem well-posed: the states $\rho_{O}$ and $\sigma_{O}\left[\Lambda \right]$ share identical operator content, confining all response to a finite physical scale rather than a formal continuum limit.

The relative-entropy Hessian (Kubo–Mori metric [59]) governs the leading near-equilibrium response on the resolved algebra, allowing the algebraically isolated tensor block to match the Einstein–Hilbert action as the lowest-order two-derivative invariant. By representing this Kubo–Mori response as a spectral trace, this framework also physically motivates the spectral-action principles of noncommutative geometry [64,65]. Rather than imposing the spectral trace as a top-down geometric postulate, it is used here as the effective matching construction for the modular Hamiltonian at the operational scale $M_{s}$.

This operational threshold defines the bandwidth below which continuum EFT and standard renormalization-group flows apply. It anchors entropic and spectral approaches to finite informational capacity. Phenomenological modified-spacetime models often introduce f(R) backgrounds or GUP-corrected tunnelling parameters to study finite-length and higher-curvature effects in black-hole thermodynamics [73,74]. The present construction is complementary but more constrained: the finite bandwidth $M_{s}$ and the reduced $R^{2}$ coefficient are fixed by boundary relative-entropy matching, rather than introduced as external f(R) or GUP parameters.

### 2.9. Time Evolution as Open Modular Dynamics and Walsh Filtration

The Hessian governs restoring forces inside a fixed diamond. Physical time evolution requires shifting the local causal workspace to the next causal diamond. Finite resolution (P5) pushes the causal-diamond tips below the regulator scale δ. Because the Hilbert space does not factorize (P1), the resolved description is expressed instead as an inclusion of accessible algebras, $A_{\mathrm{res}}\subset A_{\mathrm{full}}$.

Passing from one resolved diamond to the next coarse-grains over sub-resolution boundary data, especially near the tips. We model this update (in the Schrödinger picture) by a completely positive trace-preserving (CPTP) map [17,75] that tracks only shared observables, rendering the evolution of resolved states effectively non-unitary. In the Markovian limit, these updates approach a time-homogeneous GKSL semigroup:

$$\frac{d\rho }{d\tau }=-i\left[H_{\mathrm{eff}},\rho \right]+\sum_{a,\omega }\gamma_{a}\left(\omega \right)\left(L_{a,\omega }\rho L_{a,\omega }^{†}-\frac{1}{2}\left\{L_{a,\omega }^{†}L_{a,\omega },\rho \right\}\right),$$

where the proper time of an external observer scales as t≃δτ [76,77].

Equivalently, because the resolved state can remain correlated with inaccessible degrees of freedom, the local update admits the Kraus form

$$\Phi \left(\rho \right)=\sum_{a}K_{a}\rho K_{a}^{†},\;\sum_{a}K_{a}^{†}K_{a}=I.$$

Local irreversibility is therefore an operational consequence of finite-resolution inference, not a violation of global Wheeler–DeWitt stationarity. In this picture, “now” is the latest stable record after a CPTP update, and “time” is the irreversible sequence of such updates.

Dissipation is not introduced as an independent scale. The same Kubo–Mori kernel that governs the Hessian also sets the coarse-grained leakage, with its dimensionless coefficient fixed by the tip parameter κ [59,60]. A single finite-resolution bottleneck therefore controls both tensor stiffness and open-modular irreversibility.

A generic CPTP map does not by itself select this generation structure. The mechanism used here is more specific: graph transport on the finite boundary combined with localized pole aliasing at the six octahedral defects. This update preserves the invariant sector while damping non-invariant boundary data.

The operational update can be summarized by the axiomatic chain

$$\left(P1,P3,P5\right)\Rightarrow \rho_{\mathrm{res}}\overset{\mathrm{CPTP}}{\to }E_{\tau }=e^{\tau L}\overset{\mathrm{Markov}/\mathrm{GKSL}}{\to }A_{\mathrm{eff}}=\mathrm{Fix}\left(E_{\tau }\right)\overset{\mathrm{dephase}}{\to }G\to g\to T.$$

This chain describes the loss of accessible symmetry data under finite-resolution updating. It is retained as the coarse open-modular filtration of symmetry information. It is no longer used as a stage-counting argument for generations.

The generation-relevant structure comes from the signed boundary transport algebra. The signed octahedral transport layer carries three independent orientation signs, forming $\Gamma ≃{\left(Z_{2}\right)}^{3}$. The natural Fourier transform on this binary space is the Walsh transform. It decomposes the eight orientation modes into invariant, single-axis, two-axis and three-axis sectors, $C\left[\Gamma \right]=W_{0}⊕W_{1}⊕W_{2}⊕W_{3}$, with dimensions (1,3,3,1).

The invariant sector $W_{0}$ is the fixed sector. The generation carrier is the degree-one tangent module,

$$F_{3}\equiv W_{1}=\mathrm{span}\left\{\chi_{x},\chi_{y},\chi_{z}\right\},\;\dim F_{3}=3.$$

This is the algebraic origin of the “three”. It is not the number of stages in G→g→T; it is the dimension of the elementary tangent module selected by the signed boundary transport algebra.

The two-axis sector $W_{2}$ also has dimension three, but it represents composite two-axis data such as xy, xz and yz. It is therefore grouped with the higher multi-axis sector $W_{\geq 2}\equiv W_{2}⊕W_{3}$, giving the diagnostic filtration 1+3+4=8. The open update thus separates one fixed sector, three elementary tangent modes and four higher multi-axis modes.

Walsh sectors are distinguished dynamically by their Hamming degree *m*, the number of active transport axes. In the symmetric pole-aliasing channel, degree-*m* modes have eigenvalues $\lambda_{m}=\mu^{m}$ (0<μ<1). This establishes a strict spectral gap $\Delta_{12}=\lambda_{1}-\lambda_{2}=\mu \left(1-\mu \right)>0$ between the degree-one tangent sector $W_{1}$ and the degree-two composite sector $W_{2}$. The invariant sector $W_{0}$ remains fixed, making $W_{1}$ the least-damped non-invariant tangent sector. By contrast, the composite sector $W_{2}$ joins the damped multi-axis modes, an ordering confirmed by the finite-mesh diagnostic [61]. The generation-carrier is therefore selected as the leading non-invariant Walsh sector.

The supplementary script open_modular_lie_filtration.py [61] provides a finite computational verification of this Walsh filtration. It builds an octahedrally refined $S^{2}$ boundary mesh, evolves 8×8 orientation states under graph transport with localized pole aliasing and projects the resulting finite update into the Walsh basis.

**Figure 2:**
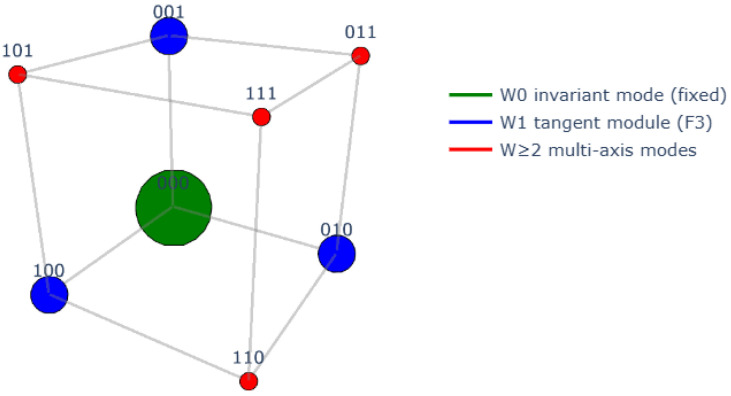
The eight orientation modes of the signed octahedral transport layer form the binary cube $\Gamma ≃{\left(Z_{2}\right)}^{3}$. This visualization is not hand-drawn; it is simulated from the finite-lattice code tracking the open-modular CPTP updates [61]. The simulation mathematically filters the surviving states into one invariant fixed mode (green), three single-axis tangent modes (blue), and four higher multi-axis modes (red), cleanly isolating the three-dimensional generation carrier as an emergent output.

Figure 2 shows the resulting diagnostic cube. It is not a hand-drawn classification, but a visualization extracted from the simulated CPTP update.

The same script also computes the Walsh survival spectrum and the ϵ=0 transport-only control (not reproduced here). The invariant mode remains fixed, the non-invariant sectors are damped with a positive spectral gap, and disabling pole aliasing keeps the non-invariant content flat. The filtration is thus driven by finite-resolution pole aliasing, not by graph transport alone.

This generation carrier also has the right spectator structure for gauge labels. For a gauge representation $V_{R}$, the matter space takes the form $F_{3}⊗V_{R}$. The gauge action acts only on $V_{R}$ and leaves the three-dimensional generation carrier untouched. Equivalently, the gauge generators commute with the projectors onto the three generation slots. The same gauge representation can therefore sit over three identical slots without inserting three independent copies by hand. In compact form, the logic is

$${\left(Z_{2}\right)}^{3}\overset{\mathrm{Walsh}}{\to }\left(1,3,3,1\right)\Rightarrow F_{3}=W_{1}\Rightarrow \dim F_{3}=3.$$

The module $F_{3}$ is the generation carrier selected by the finite boundary update.

### 2.10. Recovery of Standard Limits and Entropic Arrow of Time

**Einstein Limit.** Standard limits follow directly by varying the unified matching-scale action obtained from the relative-entropy Hessian. In the infrared ($E\ll M_{s}$), the heat-kernel expansion matches onto the Einstein–Yang–Mills–Dirac Lagrangian with the locked $\lambda =M_{P}^{2}/\left(12M_{s}^{2}\right)$ coefficient in the reduced homogeneous curvature sector; as *E* approaches $M_{s}$, the continuum EFT loses operational resolution and is replaced by discrete boundary updates. For the gravitational sector, defining the effective stress-energy tensor $T_{\mu \nu }^{\mathrm{eff}}$ via the standard metric variation of the matter action, the $R^{2}$ saturation term yields the corrected Einstein equations:

$$M_{P}^{2}G_{\mu \nu }+\frac{M_{P}^{2}}{6M_{s}^{2}}H_{\mu \nu }^{\left(R^{2}\right)}=T_{\mu \nu }^{\mathrm{eff}},\;H_{\mu \nu }^{\left(R^{2}\right)}=2RR_{\mu \nu }-\frac{1}{2}g_{\mu \nu }R^{2}-2\nabla_{\mu }\nabla_{\nu }R+2g_{\mu \nu }□R.$$

In the low-curvature regime $\left|R\right|\ll M_{s}^{2}$ the higher-order correction is heavily suppressed (by powers of $\left|R\right|/M_{s}^{2}$). Stationarity of the variational functional constrains the background to the Einstein universality class [6,66], giving $M_{P}^{2}G_{\mu \nu }\approx T_{\mu \nu }^{\mathrm{eff}}$ with stiffness set by the extensive channel capacity $N_{\mathrm{eff}}$ [37].

**Yang–Mills and Dirac Limits.** The same structure matches the Yang–Mills response, while the spinorial transport sector supplies Dirac kinematics. The vector block is fixed by the quantized WZW current algebra (P6) together with the universal modular correlator $\langle J\left(\tau \right)J\left(0\right)\rangle \propto \sin^{-2}\left(\tau /2\right)$ evaluated on the $S^{3}\times S^{1}$ history manifold; its quadratic response directly yields the gauge kinetic term and the Yang–Mills field equation $D_{\mu }F^{\mu \nu }=J^{\nu }$. Together with the scalar mass deformation, the spinorial transport block gives $(i\gamma^{\mu }D_{\mu }-m)\psi =0$. These macroscopic equations emerge jointly from the boundary response. While entropic derivations of gravity are established [6,47], this finite-resolution architecture extends that emergence to the gauge and matter sectors. The required boundary completion, discrete transport and symmetry projections block-diagonalize the leading response, yielding the Einstein, Yang–Mills and Dirac equations as a unified output rather than independent postulates.

**Hawking Scaling Limit.** At macroscopic horizon scales, the open-boundary leakage picture is compatible with the standard Hawking scaling. Distinguishing the horizon surface gravity $\kappa_{H}$ from the local tip impedance κ, one has $T_{H}=\kappa_{H}/\left(2\pi \right)$ and, for a four-dimensional Schwarzschild horizon, $A_{H}\propto \kappa_{H}^{-2}$. Hence the asymptotic thermal power scales as $P_{\infty }∼A_{H}T_{H}^{4}\propto \kappa_{H}^{2}$, up to greybody and species factors [41,78]. This shows that the finite-resolution CPTP update has the right local thermodynamic engine to reproduce the surface-gravity scaling of Hawking emission at macroscopic horizons.

**Entropic Arrow of Time.** Finite resolution (P5) supplies a structural basis for the arrow of time. Passing from one resolved diamond to the next coarse-grains over exiting data, inducing the CPTP map Φ on the resolved state $\rho_{\mathrm{res}}$. In the Markovian update rule introduced above, the thermodynamic arrow of time arises from the contractivity of relative entropy under each CPTP step [76,79], ensuring that state distinguishability decreases monotonically across successive updates. When the reference is stationary, or when the same update acts on both state and reference, contractivity makes relative entropy decrease along the coarse-grained flow:

$$S_{\mathrm{rel}}\left(\Phi \left(\rho \right)∥\Phi \left(\sigma \right)\right)\leq S_{\mathrm{rel}}\left(\rho ∥\sigma \right)\;\Rightarrow \;\langle \frac{dS_{\mathrm{rel}}}{d\tau }\rangle \leq 0.$$

As a result, the thermodynamic arrow of time (the irreversible loss of distinguishability) follows from the finite-resolution architecture and its Markovian update rule.

### 2.11. Universal Entropic Variational Principle (Conjecture)

Motivated by the construction above, we conjecture that the relative-entropy Hessian defines the emergent bulk metric under the continuum embedding. In this information-geometric picture, spacetime distance measures distinguishability: statistically similar boundary sources are geometrically close, while distinct ones are far apart. Because distinguishability is non-negative and contractive under finite-resolution coarse-graining, the emergent geometry inherits the positivity and monotonicity of the underlying Kubo–Mori metric governing linear response near equilibrium.

Identifying the relative-entropy Hessian with the Bogoliubov–Kubo–Mori monotone metric on the boundary sources Λ over $A_{O}$ gives the conjectural variational relation [79]:

$$\delta S_{\mathrm{rel}}\left(\rho_{O}∥\sigma_{O}\left[\Lambda \right]\right)=0,\;g_{AB}=\frac{1}{M_{s}^{2}}\frac{\delta^{2}S_{\mathrm{rel}}}{\delta \Lambda^{A}\delta \Lambda^{B}}{|}_{A_{O}}.$$

Here, *A* and *B* are DeWitt condensed source indices, subsuming resolved position, source type and internal tensor components into a single label. They parameterize the source space and define the target metric $g_{AB}$.

At the symmetric matched reference, this source-space metric is block-diagonal between inequivalent tensor, vector and scalar modules. This is a local tangent-space statement: away from the reference point, or at higher derivative order, suppressed off-block terms may appear as nonlinear EFT corrections.

In the macroscopic limit ($E\ll M_{s}$), the embedding maps these source directions to continuum field, tensor and gauge structures. Under this infrared mapping, the second variation reduces to the Kubo–Mori inner product on the continuous transport operator $D_{A}$. Its heat-kernel expansion then matches onto the Einstein–Yang–Mills–Dirac action, including the locked Starobinsky coefficient $\lambda =M_{P}^{2}/\left(12M_{s}^{2}\right)$, the gauge coupling $\alpha^{-1}\left(M_{s}\right)=4\pi k$ and the scalar mass deformations.

Although a computational implementation on the finite octahedral lattice is straightforward (representing $A_{O}$ as finite matrices, computing $S_{\mathrm{rel}}$ and its Hessian, solving $\delta S_{\mathrm{rel}}=0$ by iteration), the formal continuum limit remains conjectural. The extracted metric $g_{AB}$ is then coarse-grained below $M_{s}$ to recover the matched continuum EFT. A rigorous proof requires three steps: (i) an embedding map from discrete transport operators to continuum fields preserving conformal structure, (ii) infrared convergence of the discrete Hessian to the continuous heat-kernel expansion and (iii) a controlled renormalization-group flow protecting the locked coefficient $\lambda =M_{P}^{2}/\left(12M_{s}^{2}\right)$.

Until that proof is formalized, the framework operates as a bipartite effective theory: a continuous effective action below $M_{s}$ and a discrete algebraic update rule above $M_{s}$. Proving this universal conjecture forms the remaining open challenge; such a proof would establish a computationally finite, background-independent formulation of emergent semiclassical gravity.

## 3. Structural Implications at the Matching-Scale

The relative-entropy variational principle establishes a single physical matching-scale $M_{s}$ calibrated by Newton’s constant *G*. This scale is a physical threshold where the discrete boundary architecture anchors a continuum EFT description. Because the resolution is finite, the boundary measurement protocol is fixed; the vector-sector response takes a quantized (integer-level) form, with kinematic dependence determined by the modular prescription and only topological data entering the normalized susceptibility.

### 3.1. Vector-Sector Matching and Ratio Lock

On a boundary-completed algebra with fixed resolution ε=1, the gauge coupling $\alpha^{-1}\left(M_{s}\right)$ is determined by the $G_{JJ}$ block of the relative-entropy Hessian. To obtain the pixel susceptibility $\chi_{\mathrm{pix}}$, we integrate the KMS current kernel over the resolved modular window τ∈[1,2π−1]:

$$\chi_{\mathrm{pix}}=\frac{k}{4}\int_{1}^{2\pi -1}\frac{d\tau }{\sin^{2}\left(\tau /2\right)}=k\cot\left(1/2\right).$$

The kinematic factor $I_{\mathrm{mod}}=4\cot\left(1/2\right)$ is a universal constant of the matching protocol. Matching this to the canonical $-\frac{1}{4}F^{2}$ normalization fixes the coupling to the integer level k∈Z of the current algebra (P6):

$$\alpha^{-1}\left(M_{s}\right)=4\pi k.$$

Since all gauge sectors utilize the same modular protocol and $S^{2}$ flux convention, the kinematic factor $I_{\mathrm{mod}}\left(\epsilon \right)$ is universal and cancels exactly in coupling ratios. For any two sectors i,j, the matching-scale ratio is therefore strictly constrained by the integer levels, remaining independent of the modular-window convention:

$$\frac{\alpha_{i}^{-1}\left(M_{s}\right)}{\alpha_{j}^{-1}\left(M_{s}\right)}=\frac{k_{i}}{k_{j}}.$$

**Falsifiability.** Any RG-inferred matching-scale ratio incompatible with small-integer levels given the assumed boundary inventory would violate the rigidity of the protocol.

### 3.2. Modular Invariance and Weak-Mixing Angle

The boundary architecture fixes gauge response through integer current levels (P6). On the compact history manifold $S^{3}\times S^{1}$, the distinction between spatial and modular cycles is relational. The current algebra must therefore close consistently under modular exchange and under the isotropic octahedral transport rule. This closure dictates that the integer level *k* of each WZW current algebra matches the quadratic current index of the embedded matter fields. This is the two-point current normalization entering the vector susceptibility, not a separate cubic anomaly trace.

The level assignment is fixed by one object: the quadratic current index of the boundary product algebra. The trace is taken over a common boundary inventory I, with fields neutral under a given current contributing zero. For a simple factor *G*, the index is ${\mathrm{Tr}}_{I}\left(T^{a}T^{b}\right)=k_{G}\delta^{ab}$, with $k_{G}=\sum_{R}n_{R}T_{G}\left(R\right)$. For an Abelian factor, the same current metric becomes the charge-square trace, $k_{U(1)}={\mathrm{Tr}}_{I}\left(Q^{2}\right)=\sum_{i}q_{i}^{2}$. Thus, the non-Abelian and Abelian blocks are not assigned by different rules. They are the identical quadratic current normalization written in different bases: Dynkin indices for simple factors and charge squares for Abelian factors.

For $\mathrm{SU}{\left(3\right)}_{c}$, only colored quarks contribute. The six Dirac quark flavors transform in the fundamental representation. Since the color trace is already contained in $T_{\mathrm{SU}(3)}\left(3\right)=1/2$, the boundary-current index is $k_{s}=6T_{\mathrm{SU}(3)}\left(3\right)=3$. This equals the intrinsic adjoint normalization $C_{2}\left(\mathrm{adj}\right)=h_{\mathrm{SU}(3)}^{\vee }=3$, which explains the original shorthand $k_{s}=h^{\vee }$ without invoking a second prescription.

For the electromagnetic projection, the Abelian generator is *Q*. Because *Q* is color-blind, the three colors appear as an ordinary multiplicity. Expanding this trace over the explicit three-generation boundary inventory bridges the formal algebraic index directly to the specific particle content. The quarks contribute $3\;\left(\mathrm{colors}\right)\times 3\;\left(\mathrm{generations}\right)\times [{\left(2/3\right)}^{2}+{\left(-1/3\right)}^{2}]=5$, the three charged leptons contribute $3\times {\left(-1\right)}^{2}=3$ and the charged component of the Higgs doublet contributes 1. The identical quadratic metric therefore evaluates exactly to $k_{\mathrm{em}}={\mathrm{Tr}}_{I}\left(Q^{2}\right)=5+3+1=9$.

The normalized current response therefore gives the boundary ratio:

$$\frac{\alpha_{\mathrm{em}}^{-1}\left(M_{s}\right)}{\alpha_{s}^{-1}\left(M_{s}\right)}=\frac{k_{\mathrm{em}}}{k_{s}}=\frac{9}{3}=3.$$

This ratio agrees with Standard Model extrapolations to $M_{s}$ at the percent level.

Within the electroweak current block, the same normalization yields the canonical weak-mixing projection. In the selected electroweak embedding, the $SU{\left(2\right)}_{L}$ current and the canonically normalized hypercharge current share the same normalized level, $k_{1}=k_{2}$. Using the matching relation $\alpha_{i}^{-1}\left(M_{s}\right)=4\pi k_{i}$ and the standard hypercharge identity $\alpha_{\mathrm{em}}^{-1}=\alpha_{2}^{-1}+\frac{5}{3}\alpha_{1}^{-1}$, we obtain $\alpha_{\mathrm{em}}^{-1}=\frac{8}{3}\alpha_{2}^{-1}$. Inverting this ratio immediately fixes the weak mixing angle:

$$\sin^{2}\theta_{W}\left(M_{s}\right)\equiv \frac{\alpha_{\mathrm{em}}}{\alpha_{2}}=\frac{3}{8}.$$

This is a boundary-current projection, not an assumption of physical simple-group unification. It also agrees with Standard Model extrapolations to $M_{s}$ at the percent level.

**Falsifiability.** Any RG-extrapolated gauge coupling at $M_{s}$ violating the integer quantization $\alpha^{-1}\left(M_{s}\right)=4\pi k$ (for integer *k*) would falsify the discrete boundary-current architecture. Even if this quantization holds, a significant deviation from $\sin^{2}\theta_{W}\left(M_{s}\right)=3/8$ or $\alpha_{\mathrm{em}}^{-1}\left(M_{s}\right)/\alpha_{s}^{-1}\left(M_{s}\right)=3$ beyond ordinary RG would rule out the minimal faithful embedding of the matter inventory.

### 3.3. Symmetry Filtration and Matter-Generation Carrier

Open modular dynamics irreversibly filter phase-sensitive information on the resolved boundary algebra. As established in Section 2, the Walsh filtration isolates a three-dimensional tangent module,

$$F_{3}=\mathrm{span}\left\{\chi_{x},\chi_{y},\chi_{z}\right\},$$

inside the signed boundary transport algebra. In this picture, flavor is not an added copy structure, but a symmetry-filtration effect driven by finite resolution.

Intuitively, the eight orientation states form the corners of a binary cube. The Walsh transform acts as the discrete Fourier transform of this cube, rewriting local corner labels into global modes: one constant mode, three single-axis modes and four multi-axis modes (such as xy, xz or xyz). The three single-axis modes are the elementary tangent directions of the finite transport algebra. The relevant “three” is therefore not a temporal stage count, but the exact dimension of the primary Walsh tangent module selected by the boundary update.

The framework thus supplies three generation slots natively. For a gauge representation $V_{R}$, the spectator matter-generation carrier $F_{3}⊗V_{R}$ confines the gauge action to $V_{R}$, leaving the generation space untouched. The same gauge representation therefore sits symmetrically over the three slots, so the threefold carrier behaves as one structured generation space rather than three unrelated sectors.

The open-modular Walsh filtration supplies a dynamically selected three-dimensional matter-generation carrier. Identifying it with the full observed Standard Model flavor sector would require extending the analysis to chirality, Yukawa couplings, mixing and mass hierarchy.

**Falsifiability.** The mechanism fails if the open-modular update does not isolate the degree-one Walsh module, if the ϵ=0 control contracts non-invariant data, or if pole aliasing fails to drive the filtration. Furthermore, because the selected Walsh tangent carrier is strictly three-dimensional, a definitive fourth chiral Standard Model generation would falsify this minimal framework.

### 3.4. Semiclassical Entropy Consistency

The same Hessian-to-spectral map also passes the standard horizon-entropy check. For a horizon of area *A*, the leading boundary record count over one modular cycle gives:

$$S_{0}\left(A\right)=2\pi \left(AM_{s}^{2}\right)N_{\mathrm{eff}}=\frac{A}{4G},$$

using the constitutive relation $M_{P}^{2}=N_{\mathrm{eff}}M_{s}^{2}$. Thus, the finite-resolution screen reproduces the Bekenstein–Hawking area law rather than replacing it. The spectral expansion then takes the standard form:

$$S\left(A\right)=\frac{A}{4G}+\alpha \ln\;\left(\frac{A}{L_{s}^{2}}\right)+\beta \frac{L_{s}^{2}}{A}+O\;\left(\frac{L_{s}^{4}}{A^{2}}\right).$$

The logarithmic term belongs to the usual semiclassical correction class, while the router-specific finite-resolution signatures first enter through power-suppressed terms. The architecture therefore preserves the macroscopic semiclassical entropy structure and confines its distinctive discrete effects to the $O(L_{s}^{2}/A)$ regime.

**Falsifiability.** The finite transport layer fails this semiclassical check if it modifies the leading A/4G law or produces router-specific corrections at leading or logarithmic order.

### 3.5. Geometric Coherence and $R^{2}$ Spectral Plateau

The relative-entropy Hessian isolates $R^{2}$ as the leading four-derivative correction at the matching scale $M_{s}$, with coefficient $\lambda =M_{P}^{2}/\left(12M_{s}^{2}\right)$ fixed by the ratio of heat-kernel coefficients on the modular manifold (Section 2). This identification places high-curvature dynamics in the plateau universality class [40].

The coherence duration $N_{*}$ is derived from open modular dynamics. Let $\tau_{\mathrm{coh}}$ denote the resolved modular time over which the scalar-curvature mode remains phase-coherent under repeated CPTP updates. The finite-resolution tips generate an irreducible distinguishability increment fixed by the tip-defect parameter κ=1/(6π). For the scalar-curvature projection, this increment is distributed over the two tip bottlenecks and the D=3 independent spatial transport axes. The projected increment per resolved modular update is therefore κ/(2D), giving the first-passage time $\tau_{\mathrm{coh}}=2D/\kappa$.

Physical expansion time is obtained from modular time through dN=Hδdτ. On the $R^{2}$ plateau, $M_{R}=M_{s}=\delta^{-1}$ and $H≃M_{R}/2$, so Hδ≃1/2. The coherence duration in e-folds is therefore:

$$N_{*}=H\delta \;\tau_{\mathrm{coh}}=\frac{1}{2}\frac{2D}{\kappa }=\frac{D}{\kappa }=18\pi \approx 56.55.$$

This result has a direct geometric reading. Since $\kappa^{-1}=3\times 2\pi$, the dynamical expression becomes:

$$N_{*}=\frac{D}{\kappa }=3\;\left(\mathrm{axes}\right)\times 2\pi \;(\mathrm{modular}\;\mathrm{cycle})\times 3\;(\mathrm{inverse}\;\mathrm{tip}\;\mathrm{fraction})=18\pi .$$

Thus, the router counting is not an independent postulate. It is the discrete-geometric form of the same first-passage result. The eight-fold orientation redundancy used in the Walsh-module filtration does not enter this coherence-time derivation. The plateau coherence time is therefore fixed by the same tip-defect calibration that controls finite-resolution modular leakage.

On this plateau, the spectral tilt and tensor-to-scalar ratio become:

$$n_{s}=1-\frac{2}{N_{*}}\approx 0.9646,\;r=\frac{12}{N_{*}^{2}}\approx 0.0038.$$

The predicted scalar tilt lies close to the Planck value, while the tensor ratio remains below current bounds and provides a sharp target for next-generation CMB B-mode searches.

These shape observables follow a geometric operational chain, translating the resolution limit into a macroscopic coherence time (independently of boundary capacity):

$$\kappa \to \tau_{\mathrm{coh}}\to N_{*}\to \left(n_{s},r\right).$$

Finite *N* also fixes the normalization of the plateau fluctuations. This is not an additional phenomenological fit, as *N* is already fixed by the boundary-capacity construction in Section 1. In the selected $R^{2}$ plateau sector, the scalaron scale is $M_{R}=M_{s}$, while the Newton calibration gives $M_{P}^{2}=\kappa NM_{s}^{2}$. Using κ=1/(6π) and $N_{*}=18\pi$, the scalar amplitude becomes:

$$A_{s}=\frac{M_{R}^{2}N_{*}^{2}}{24\pi^{2}M_{P}^{2}}=\frac{N_{*}^{2}}{24\pi^{2}\kappa N}=\frac{81\pi }{N}≃2.1\times 10^{-9}.$$

This predicted scalar amplitude lies close to the observed CMB scalar-ampli- tude normalization.

Unlike the spectral tilt, this normalization introduces a distinct, parallel operational chain that explicitly relies on the finite network capacity:

$$\left(N,\kappa ,N_{*}\right)\to A_{s}.$$

Here the absolute Newton calibration cancels. The scalar amplitude is therefore a downstream capacity check, not an input used to define $N_{*}$. In the formal infinite-capacity limit N→∞, the scalar signal would vanish; for substantially smaller *N*, it would be overproduced. This makes $A_{s}\propto N^{-1}$ the most direct macroscopic imprint of finite boundary capacity in the plateau sector.

**Falsifiability.** The selected plateau sector fixes κ, *N* and $N_{*}$ before comparison with cosmological data, leaving no free inflationary parameter. These predictions align closely with Planck 2018 estimates for $n_{s}$ and $A_{s}$, while $r≃3.8\times 10^{-3}$ provides a sharp target for next-generation B-mode experiments. Any robust observational deviation from this linked $\left(n_{s},r,A_{s}\right)$ triad would falsify the selected minimal boundary model and its $R^{2}$ plateau matching.

## 4. Conclusions and Outlook

This work reframes semiclassical gravity as local statistical inference on a fixed, finite-resolution boundary algebra. The foundational axioms (P1–P7) provide the minimal ingredients for subsystems, clocks and observables in a background-independent setting. Here, finite resolution is not just a technical regulator; it is the physical condition that makes the relative-entropy variational principle well posed.

The novelty lies in combining established tools (causal diamonds, modular flow, relative entropy, effective field theory) into a single finite-resolution inference architecture. Boundary completion fixes the algebra, relative entropy provides the variational principle, the Kubo–Mori Hessian sets the response metric and CPTP updates drive time evolution. Together, these elements generate linked outputs rather than independent assumptions: sectoral Hessian decoupling, a unified matching scale, integer-level gauge response, plateau amplitudes and the open-modular Walsh filtration.

The first structural payoff is a finite internal response capacity. The boundary topology, tip bottleneck and spinorial twist fix the channel count to

$$N=\sqrt{2}\exp\left(8\pi +\frac{1}{6\pi }\right)\approx 1.23\times 10^{11}.$$

Calibrating the coherent fraction of these channels to Newton’s constant then sets the matching scale,

$$M_{s}\approx 3.02\times 10^{13}\;\mathrm{GeV}.$$

Instead of postulating a graviton or a new bulk field, the framework starts with a finite boundary inference problem. Gravity is identified as the extensive tensor stiffness of these coherent response channels.

At the matching scale, the relative-entropy Hessian natively separates into tensor, vector and scalar blocks. This structure is not added by hand; it follows directly from the boundary symmetries. The tensor block yields Einstein stiffness and the $R^{2}$ plateau, the vector block dictates integer-level Yang–Mills response and the scalar/spinorial blocks supply leading mass and Dirac structures. This same mapping also organizes horizon entropy.

This creates a direct bridge between semiclassical gravity and quantum fields. Rather than quantizing gravity by adding microscopic degrees of freedom, the framework derives the continuum geometry and matter response inferred by a finite boundary algebra under relative-entropy minimization. The output is tightly constrained: Einstein–Yang–Mills–Dirac structure, a locked $R^{2}$ coefficient, integer current levels and open modular evolution all emerge from the same architecture.

Two structural consequences stand out. First, tensor response is extensive, scaling with the number of coherent channels, while vector and scalar responses are intensive. This explains why gravity is collectively stiff while gauge couplings are normalized per channel. Second, time evolution between causal diamonds is intrinsically open. Repeated CPTP updates filter boundary data into stable commuting sectors, isolating a three-dimensional matter-generation carrier. The threefold carrier is dynamically selected, not inserted by hand; completing the Standard Model flavor map requires extending the analysis to chirality, Yukawa couplings, mixing and mass hierarchy.

The framework also delivers concrete observational targets. The modular coherence time fixes the spectral tilt ($n_{s}≃0.965$) and tensor-to-scalar ratio (r≃0.0038), while boundary capacity dictates the scalar fluctuation amplitude ($A_{s}≃2.1\times 10^{-9}$). We do not dial these parameters independently; they flow from the same finite-capacity geometry that fixes the matching scale. Any robust deviation from this triad would falsify the minimal boundary sector.

A strict input → output hierarchy governs the entire framework. The axioms define the inference problem, the minimal sector fixes the topology and transport and Newton’s constant provides the sole dimensional calibration. Everything else (channel capacity, Hessian structure, gauge levels, plateau coefficients, scalar amplitudes) arises as a constrained output. We are not fitting phenomenological parameters; we are testing the consequences of a fixed boundary architecture.

Finally, a supplementary simulation toolbox makes this operational engine executable [61] (a user guide is provided as File S1). By building octahedrally refined $S^{2}$ boundary meshes, dense Hessian matrices, variational solvers and CPTP update maps, these scripts provide finite-dimensional checks of Hessian decoupling, quadratic inference and Walsh filtration. They demonstrate that the proposed mechanisms can be cleanly constructed, inspected and falsified computationally on explicit finite meshes.

The framework rests on a finite analytic core with computable representatives and falsifiable predictions. We have shown that semiclassical gravity, gauge response and the proposed matter-generation carrier structure can be organized as local inference on a finite-resolution boundary algebra. The main task ahead is to formalize and demonstrate the conjectured Universal Entropic Variational Principle.

Technical details and exploratory materials are provided in the companion note [80].

## Data Availability

The original contributions presented in this study are included in the article. Further inquiries can be directed to the corresponding author.

## Supplementary Materials

The following supporting information can be downloaded at: https://www.mdpi.com/article/10.3390/e28060606/s1, File S1: Supplementary Simulation Toolbox Guide. The complete simulation repository, including the Python source code and interactive HTML outputs, is hosted on Zenodo [61].
