# Supplementary material for: Relative-Entropy Variational Principle for Semiclassical Gravity with Finite-Resolution Boundaries
Source: Entropy (Basel). 2026 May 28;28(6):606. doi: 10.3390/e28060606 (PMC13298078; doi:10.3390/e28060606)
Supplement: Supplementary file 1 [file entropy-28-00606-s001.zip › entropy-4230566-supplementary.pdf]

Relative-Entropy Variational Principle for Semiclassical Gravity with Finite-Resolution Boundaries, O. Nusbaumer, 2026

Supplementary Simulation Toolbox Guide

This supplementary toolbox provides a computational companion to the paper. Rather than simulating the full continuum theory, it tests explicit finite-dimensional representatives of the core mechanisms. Every simulation is grounded in a dynamically computed boundary—a triangulated S^2 mesh built via repeated octahedral refinement. Vertices, graph adjacency, transport operators, and update maps are derived directly from this geometry, ensuring all outputs are rigorous numerical results rather than hardcoded illustrations.

The toolbox contains three standalone scripts:

`hessian_sector_decoupling.py` verifies the algebraic origin of the tensor/vector/scalar Hessian decomposition. By applying an octahedral symmetry projection to a fully mixed local response, it proves that the block-diagonal structure is strictly dictated by the finite boundary architecture, not imposed by ansatz.

`variational_inference_dynamics.py` validates the fixed-diamond quadratic inference problem. It computes the finite inference problem directly on the boundary graph, confirming through conjugate-gradient tracking and 3D relaxation animations that the proxy is strictly convex, numerically stable, and spatially interpretable.

`open_modular_lie_filtration.py` tests the open-modular update mechanism. It executes a completely positive trace-preserving (CPTP) map using graph transport and localized pole-aliasing. Strict matrix audits and a Walsh-mode diagnostic track exactly how repeated updates irreversibly filter non-invariant boundary data.

Together, these scripts operationalize the framework's analytic arguments. They demonstrate that the proposed finite-resolution architecture is mathematically sound, computationally explicit, and strictly falsifiable.

Table of figures:

Figure S1: Generic Hessian before symmetry projection
Figure S2: Symmetry-projected trace-free tensor/vector/scalar Hessian
Figure S3: Raw tensor control with trace-scalar coupling
Figure S4: Broken-symmetry control
Figure S5: Leakage under controlled contamination
Figure S6: Spatial purity under CPTP polar aliasing
Figure S7: Walsh-module survival spectrum 1 + 3 + 4
Figure S8: Walsh diagnostic cube: 1 + 3 + 4 module classes
Figure S9: Filtration weight and epsilon = 0 control (non-invariant distinguishability contraction)
Figure S10: Topological rigidity: coordination defects on the finite S^2 router
Figure S11: Localized inference and sector-silence certificates
Figure S12: Quadratic inference convergence on the finite boundary

hessian_sector_decoupling.py

This script tests whether the relative-entropy Hessian natively separates into tensor, vector, and scalar sectors. The computation begins with a generic, dense local response that explicitly mixes all source components. This matrix is coupled to the S^2 mesh via the graph transport operator. Applying the octahedral symmetry projection then eliminates forbidden mixed channels. This proves the sector split is an inherent consequence of the finite boundary symmetry, rather than a hand-inserted block structure.

The script produces one html dashboard:

- hessian_sector_decoupling.html

hessian_sector_decoupling.html

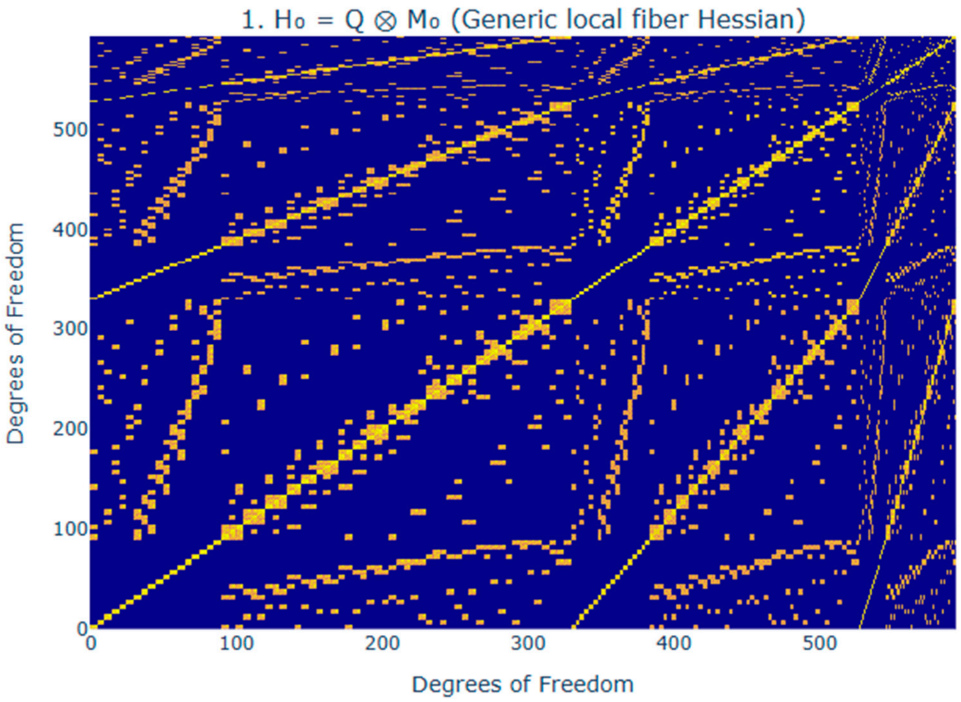

Figure S1: Generic Hessian before symmetry projection

The starting matrix is dense, with tensor, vector, and scalar components visibly coupled. This confirms the computation does not assume the final decoupled state.

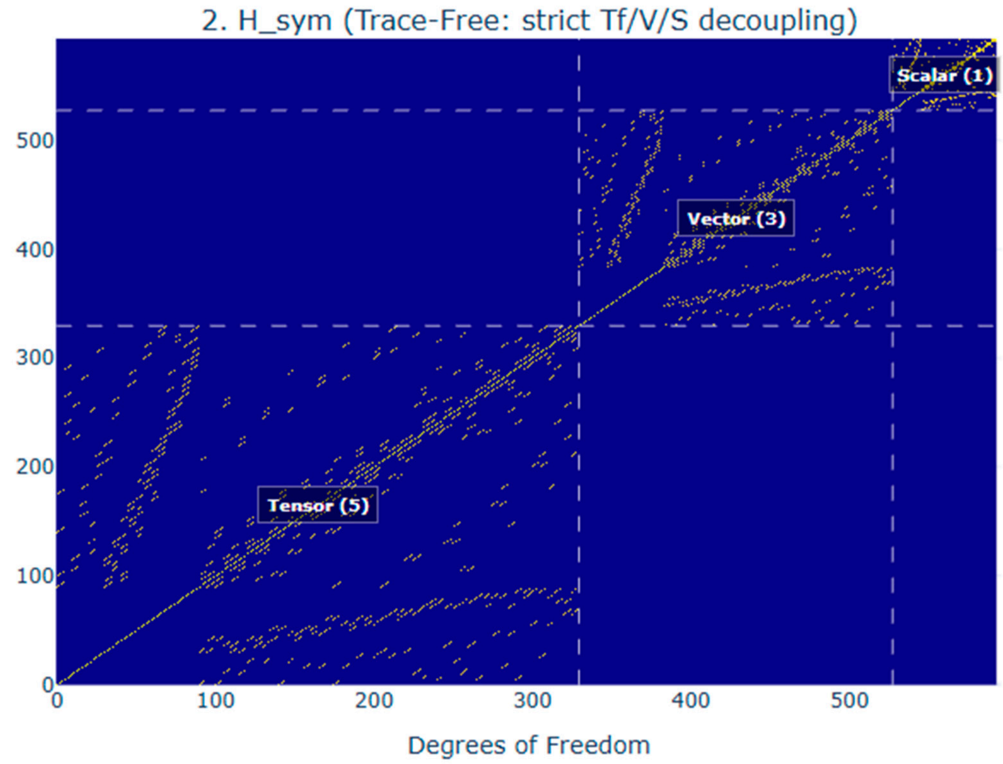

Figure S2: Symmetry-projected trace-free tensor/vector/scalar Hessian

Following the symmetry projection, the Hessian orthogonalizes into three distinct diagonal blocks. Off-block terms vanish to numerical precision. Tensor, vector, and scalar perturbations now produce strictly isolated responses. This confirms that finite boundary symmetry alone is sufficient to isolate the independent response sectors.

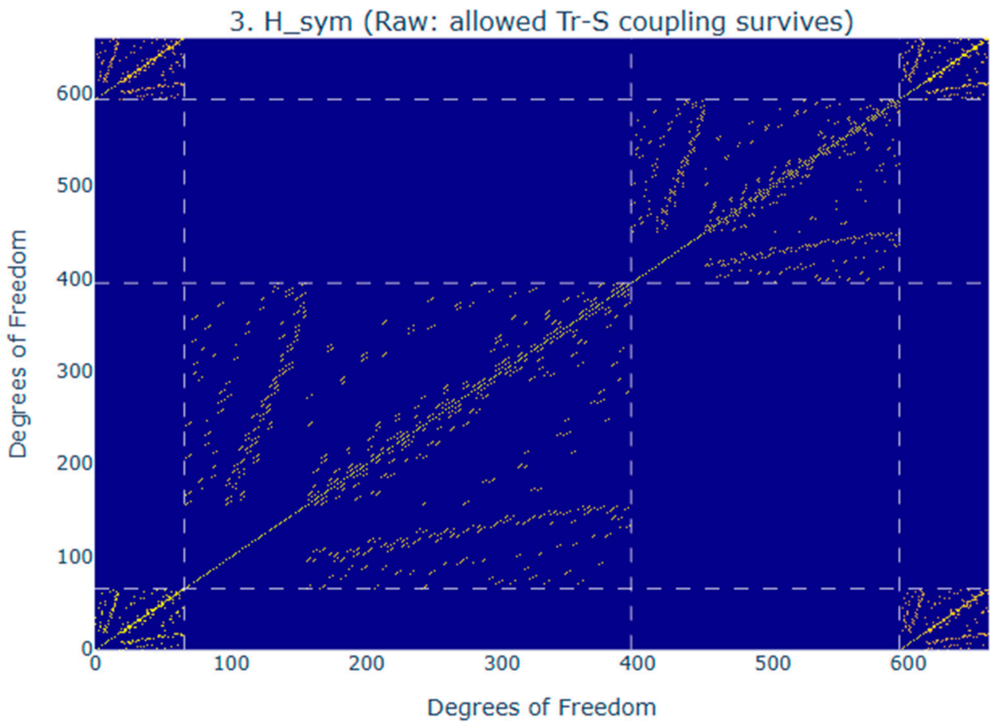

Figure S3: Raw tensor control with trace-scalar coupling

Retaining the tensor trace introduces a valid trace-scalar coupling, which behaves as an allowed scalar and correctly survives the projection. Forbidden trace-free and tensor/vector couplings still cleanly vanish. This confirms the operation accurately filters only the channels explicitly forbidden by symmetry.

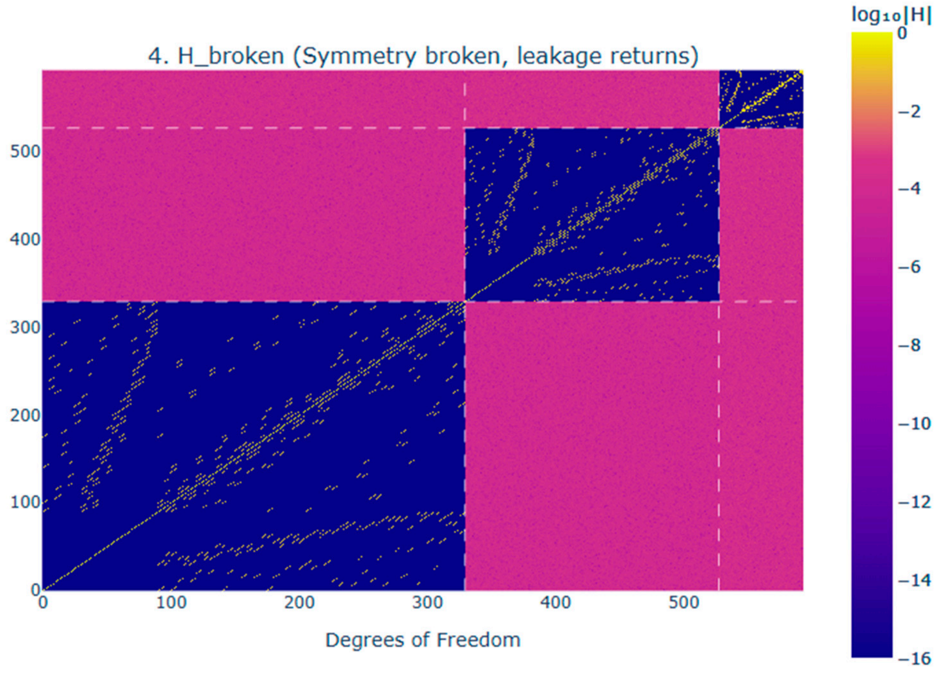

Figure S4: Broken-symmetry control

Injecting artificial off-block noise immediately restores forbidden cross-sector terms. This verifies diagnostic sensitivity, proving the clean sector split is a genuine algebraic result rather than a plotting artifact.

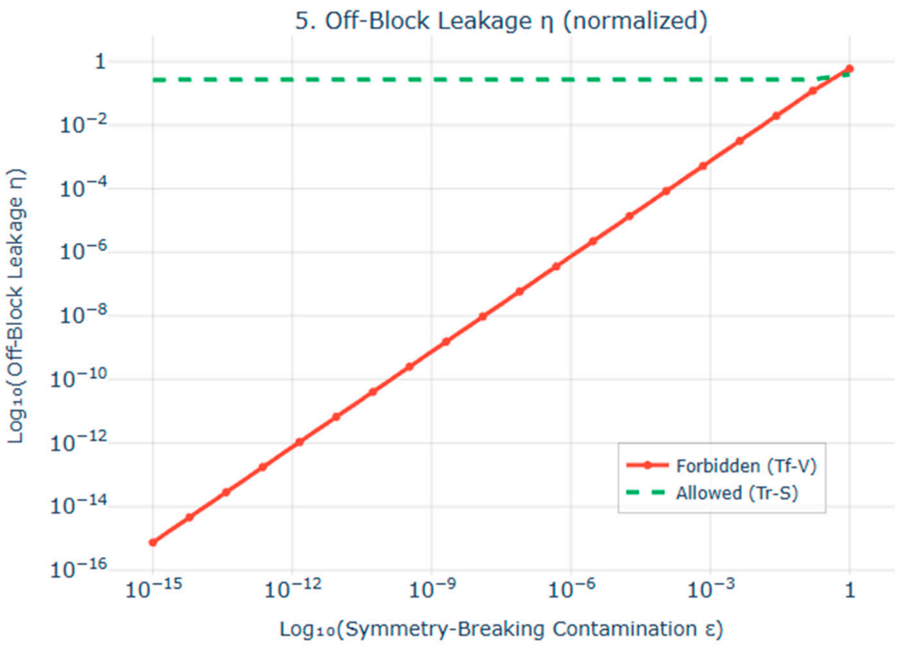

Figure S5: Leakage under controlled contamination

Tracking cross-sector leakage against increasing artificial contamination shows forbidden leakage scaling accordingly, while the allowed trace-scalar channel maintains its correct baseline. This falsifiability test confirms that sector isolation requires unbroken finite symmetry.

open_modular_lie_filtration.py

This script tests the open modular update mechanism on the octahedrally refined S^2 boundary. By applying a completely positive trace-preserving (CPTP) map—combining graph transport with localized metric aliasing at the six pole defects—it tracks the filtration of local orientation states from explicit mesh dynamics rather than theoretical approximations.

The script produces two HTML dashboards:

- open_modular_lie_filtration.html
- open_modular_purity_flow_animation.html)

open_modular_lie_filtration.html

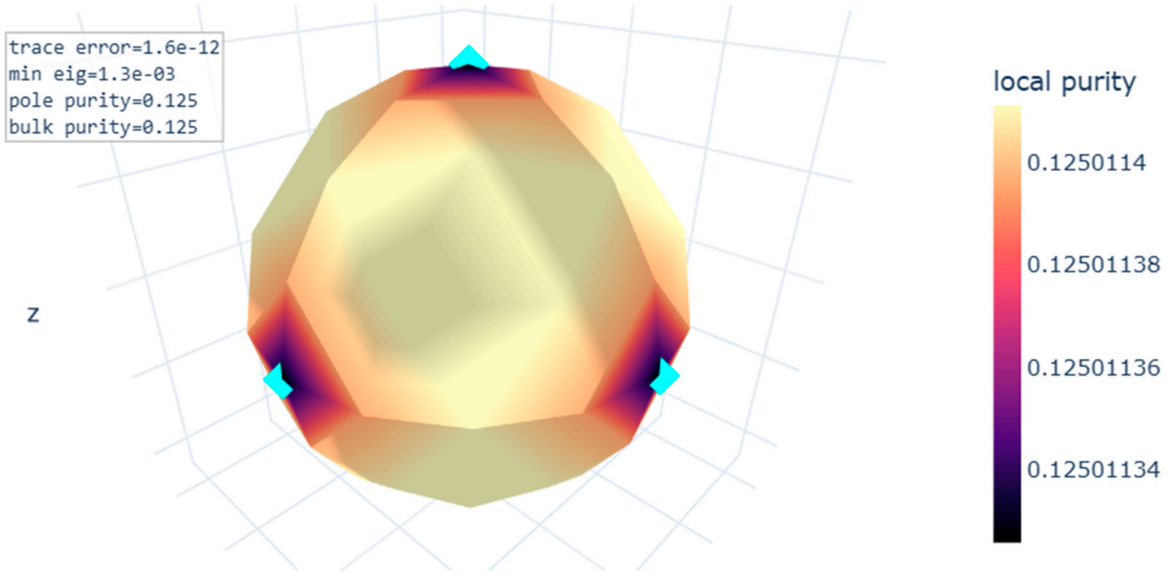

Figure S6: Spatial purity under CPTP polar aliasing

This panel shows the 3D purity map on the finite boundary after the open modular update. The strongest purity loss appears at the six octahedral poles and then spreads into the bulk through transport. This is the direct spatial signature of the mechanism. It shows that the poles are not decorative mesh features. They are the localized sites where unresolved information leaves the resolved description. This is a strong validation because it ties the filtration mechanism to an explicit geometry. The effect is computed on the finite mesh and appears at the expected topological defects.

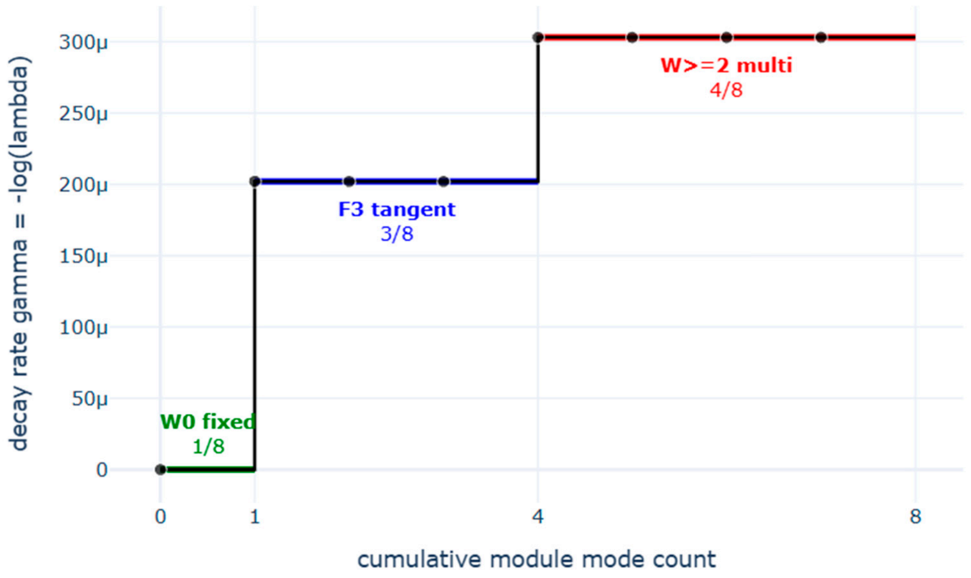

Figure S7: Walsh-module survival spectrum 1 + 3 + 4

This panel shows the decay strengths of the eight Walsh modes under the projected update. These are computed directly from the effective finite update map. The result is not a featureless decay. The modes split into a clear $1 + 3 + 4$pattern: one invariant mode, three more robust single-axis modes, and four faster-decaying multi-axis modes. This matters because it shows that the finite geometry filters boundary data in a structured way. It does not simply erase all information uniformly. It produces a clear hierarchy of mode survival. This is a strong validation because it tests the central claim at the level of the update spectrum itself. The hierarchy is an output of the computation.

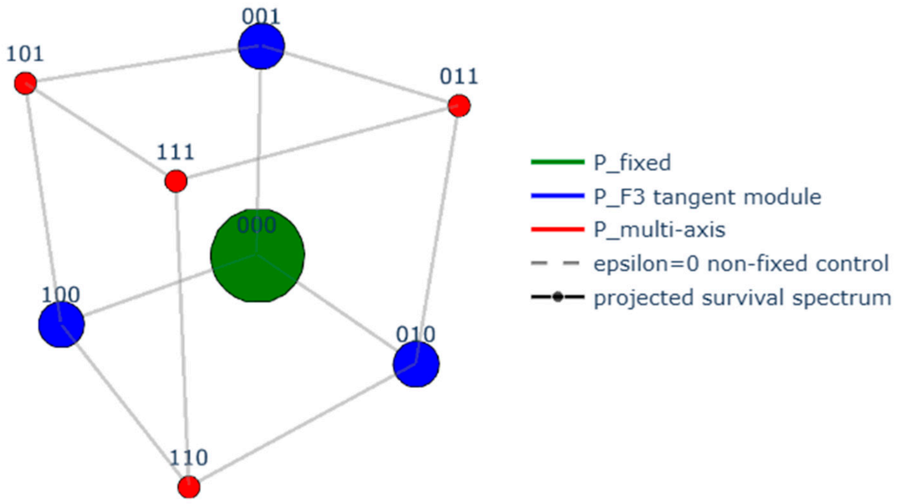

Figure S8: Walsh diagnostic cube: 1 + 3 + 4 module classes

This panel gives a 3D view of the finite diagnostic state space associated with the eight Walsh modes. Its role is interpretive. It shows how the eight modes are organized and why the $1 + 3 +$ 4grouping is natural in the finite boundary setting. It makes the distinction between invariant, single-axis and multi-axis sectors visually intuitive. This is a useful validation tool because it helps connect the spectral result of Figure S12 to a concrete state-space picture. It shows that the mode hierarchy is not just a list of numbers, but a structured geometric organization.

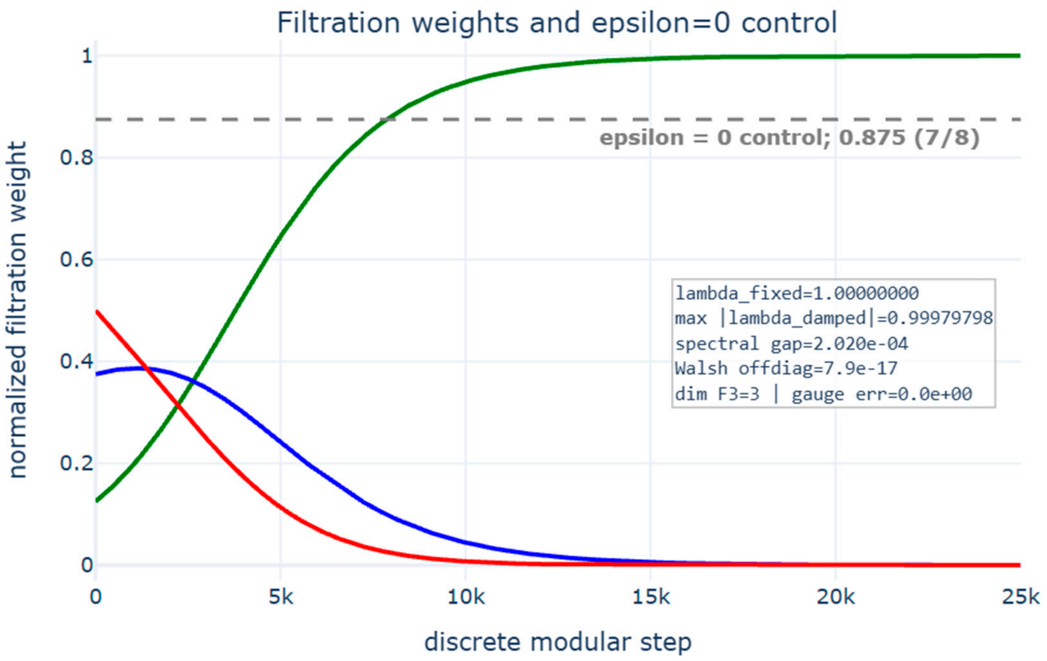

Figure S9: Filtration weight and epsilon = 0 control (non-invariant distinguishability contraction)

This panel shows how the total non-invariant mode power contracts under repeated CPTP updates. It also includes the epsilon = 0 control, where pole aliasing is switched off and only transport remains. With aliasing active, non-invariant power decays clearly over time. With epsilon = 0, the control stays essentially flat.

This is the sharpest falsification test in the script. It shows that the arrow of time in the model is not caused by background transport alone. It is generated by finite-resolution coarse-graining at the poles.

Together, these four figures test the mechanism from complementary angles: spatially, spectrally and dynamically. They show where the filtering occurs, which modes survive longest, how the mode hierarchy is organized, and why the contraction is genuinely caused by the pole-aliasing channel. This is exactly the right way to validate the theory computationally, because it checks the mechanism itself rather than only its final consequence.

open_modular_purity_flow_animation.html

This dashboard contains an interactive 3D animation of the purity field on the spherical boundary. The mesh is displayed in 3D, and each node is colored by its local purity. The animation shows how this purity changes step by step under repeated CPTP updates.

At each update, the state is transported across the mesh and partially aliased at the six pole defects. The animation tracks how the local state becomes less pure, where this first happens, and how the effect spreads across the boundary.

The loss of resolved information does not happen uniformly. It starts at the poles, which act as localized bottlenecks of the finite-resolution boundary, and then spreads through transport over the mesh. This is the concrete finite version of open modular filtration.

This animation makes the mechanism immediately visible. One can directly see that the poles are operationally active and that the dynamics are spatially structured. This is important because

the theory does not describe time evolution as an abstract matrix effect, but as an open update on a resolved boundary layer. The animation shows exactly that.

variational_inference_dynamics.py

This script tests the finite quadratic inference problem on an explicit resolved boundary.

It is complementary to hessian_sector_decoupling.py. The Hessian-sector script tests why the tensor, vector and scalar sectors decouple. This script starts from the selected block-decomposed quadratic proxy and tests whether the resulting inference problem is numerically well-posed, spatially local and visually interpretable.

The boundary is an octahedrally refined finite-element-style triangulated S^2 mesh. The code builds the vertices, faces, adjacency matrix, graph Laplacian and transport operator directly from the mesh. It then solves the finite inference problem on that graph. The outputs are computed from the mesh and the linear-algebra problem. They are not hard-coded illustrations of the expected result.

The script produces three HTML dashboards:

- topology_response.html: finite boundary topology and localized tensor response
- relaxation_animation.html: animated diamond relaxation toward the inferred solution.
- quadratic_inference_convergence.html: solver convergence and numerical solvability certificate.

topology_response.html

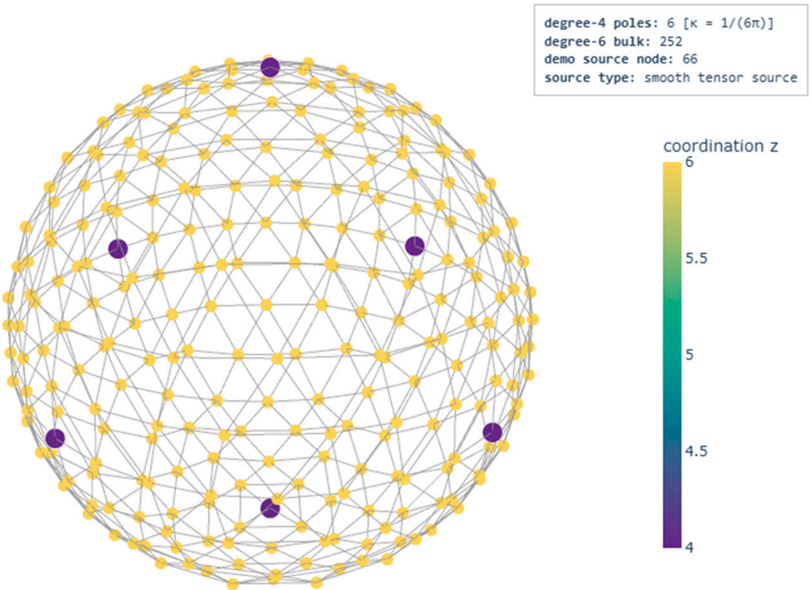

Figure S10: Topological rigidity: coordination defects on the finite S^2 router

This figure shows the resolved spherical boundary mesh generated by repeated octahedral bisection. Most nodes are regular degree-six bulk nodes. Six nodes remain degree-four. These are the stable octahedral pole defects. The mesh also satisfies the closed-sphere topology check. This matters because the theory does not use a featureless continuum boundary. It uses a resolved transport layer with fixed topology. The plot verifies that the numerical boundary has the

intended S^2topology, the intended octahedral structure and the six persistent pole defects required by the finite-resolution architecture. This is a useful verification because all later transport and inference results are built on this same graph. The figure shows the actual computational scaffold.

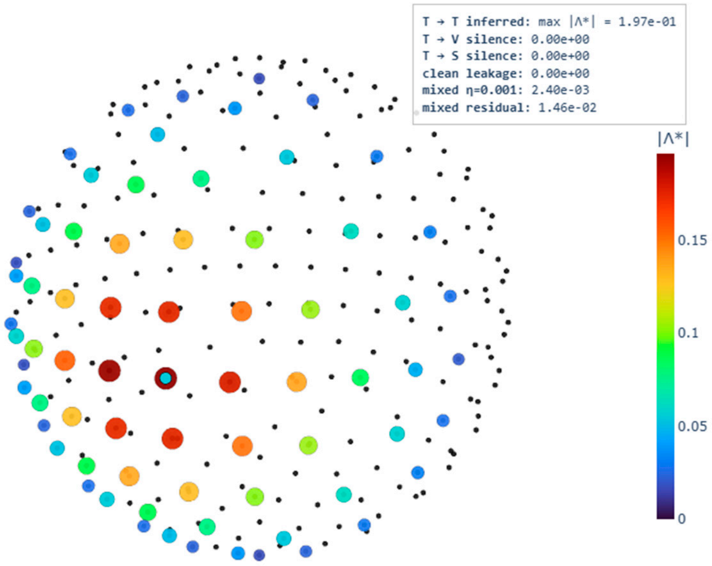

Figure S11: Localized inference and sector-silence certificates

This shows the response to a smooth localized tensor source placed on a regular bulk node of the same boundary mesh. The source is not a singular point spike. It is a smoothly resolved deformation on the finite sphere. The code solves the quadratic inference problem and displays the inferred tensor response. The response remains localized but spreads through the graph, as expected from a finite stiffness operator. The certificate reports that the vector and scalar sectors remain silent to numerical precision for this selected proxy. A tensor source produces a tensor response without spurious gauge or scalar excitation. This matters because it turns the matrix factorization into a spatial statement. Sector separation is not only visible in an abstract block matrix. It also holds for a localized deformation on the finite boundary. This is a strong verification because it checks the mechanism in the geometry where the theory is meant to operate: a resolved causal-diamond boundary with graph-local transport.

relaxation_animation.html

This HTML contains an interactive animation of the fixed-diamond variational relaxation. The animation shows the tensor field during gradient descent toward the stationary inferred solution. The left sphere displays the current inferred tensor field. The right sphere displays the remaining distance to the converged stationary field. Each frame advances the same descent trajectory used in the convergence dashboard. The field starts away from the optimum and relaxes smoothly through the graph-Hessian stiffness. The displayed title tracks the gradient step, the relative gap, the residual and the remaining mismatch.

The relative-entropy variational principle is not just a static algebraic condition. In the finite quadratic approximation, it defines a concrete relaxation path toward the best-matched boundary configuration.

This is fixed-diamond relaxation. It is not physical time evolution between different causal diamonds. It visualizes the internal inference process on one fixed boundary algebra.

The animation is useful because it links the solver certificate to the spatial geometry. The same mesh, source, Hessian proxy and descent history are used in the graph and in the animation. The viewer sees that the minimization is smooth, local and mesh-driven. The field does not jump arbitrarily. It relaxes through the finite boundary stiffness encoded by the graph operator.

quadratic_inference_convergence.html

This dashboard contains one main convergence figure with a numerical certificate. It is one of the most important verification outputs of the toolbox.

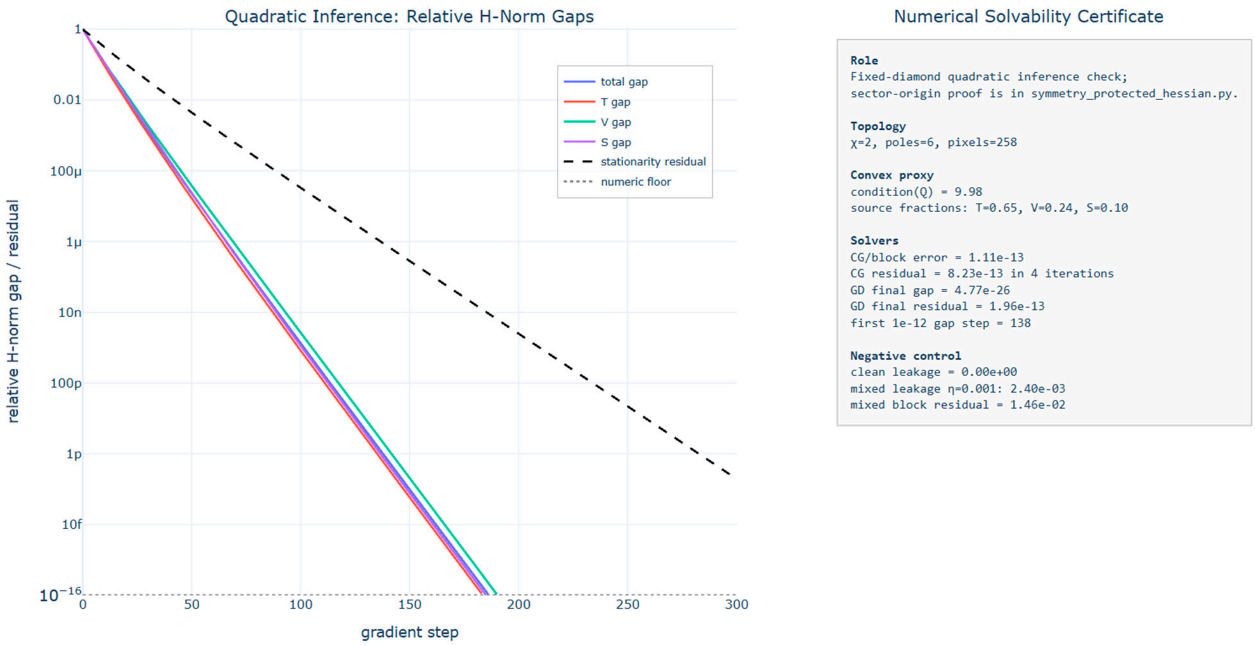

Figure S12: Quadratic inference convergence on the finite boundary

These figures track the relative variational gap and stationarity residual during gradient descent. The total gap, alongside the independent tensor, vector, and scalar components, decreases cleanly on a logarithmic scale. This confirms the selected finite proxy operates as a strictly convex inference problem, relaxing consistently toward a unique stationary solution without numerical instability. The certificate panel reports the underlying numerical validations:

- ✓ **Mesh integrity:** The topology remains closed with exactly six octahedral pole defects.
- ✓ **Operator stability:** The graph operator is properly conditioned for stable inference.
- ✓ **Comprehensive probing:** The smooth source excites all three response sectors simultaneously, ensuring the full operational capacity is tested.
- ✓ **Solver agreement:** The global conjugate-gradient solution matches the independent blockwise solution to numerical precision.
- ✓ **Convergence:** Gradient descent successfully reaches the target gap and residual.
- ✓ **Falsifiability control:** The clean proxy maintains negligible cross-sector leakage. However, introducing artificial off-block mixing predictably breaks the blockwise

factorization, causing a detectable residual spike. This proves the diagnostic is strictly sensitive to broken sector symmetry.

This computation demonstrates that the relative-entropy Hessian defines a well-posed, explicitly solvable local inference problem on the finite boundary algebra. By verifying global optimization, sector factorization, and negative controls, it validates the variational mechanics directly from first principles without tuning phenomenological parameters.

Conclusions

The simulation scripts provide an independent computational validation of the paper's finite-boundary architecture. Rather than hardcoding expected outcomes, they explicitly construct the resolved causal-diamond boundary (with its closed S^2 topology, graph-local transport, and finite source fibers) and verify the framework's core mechanisms from first principles.

Symmetry-Protected Sector Decoupling (`hessian_sector_decoupling.py`)

This script demonstrates that the separation of the Hessian into orthogonal tensor, vector, and scalar blocks is a strict algebraic consequence of the finite boundary symmetry. Applying the octahedral symmetry projection to a fully mixed response matrix cleanly isolates the allowed sectors to numerical precision. Built-in controls verify this selection rule: allowed trace-scalar mixing naturally survives, while deliberately injected forbidden leakage is instantly detected.

Stable Variational Inference (`variational_inference_dynamics.py`)

This script confirms that the relative-entropy proxy defines a well-posed, computationally stable inference problem on the finite boundary algebra. The global conjugate-gradient solution exactly matches the independent blockwise solution, demonstrating strictly convex gradient descent. Spatially, it illustrates how a localized tensor source relaxes smoothly across the boundary graph without artificially exciting the silent vector and scalar sectors.

Open-Modular Filtration (`open_modular_lie_filtration.py`)

This script isolates the physical engine of irreversibility: metric aliasing at the six octahedral pole defects. By auditing the completely positive trace-preserving (CPTP) updates, the simulation proves that non-invariant data contracts *only* when polar aliasing is active; graph transport alone (epsilon=0) preserves the information. Furthermore, the Walsh diagnostic reveals a strict 1+3+4 survival hierarchy (one invariant, three single-axis, and four multi-axis modes), showing exactly how the discrete update sorts boundary data into stable and decaying classes.

Taken together, the toolbox validates the operational core of the framework without relying on tuned phenomenological parameters. The explicit numerical representatives prove that the proposed axiomatic structure inherently generates orthogonal response sectors, stable fixed-diamond inference, and irreversible modular filtration.
